# Effects of vaping on physical and mental health in at-risk populations (VAPE): mixed-methods study of motivations for and perspectives on vaping in patients with opioid use disorder

**DOI:** 10.1192/bjo.2025.6

**Published:** 2025-04-02

**Authors:** Alessia D’Elia, Balpreet Panesar, Nitika Sanger, Laura O’Neill, Tea Rosic, Leonora Regenstreif, Kevin Park, Claire de Oliveira, David C. Marsh, Luciano Minuzzi, Lehana Thabane, Zainab Samaan

**Affiliations:** Neuroscience Graduate Program, McMaster University, Hamilton, Ontario, Canada; Department of Psychiatry and Behavioural Neurosciences, St Joseph’s Healthcare Hamilton, Hamilton, Ontario, Canada; Medical Science Program, McMaster University, Hamilton, Ontario, Canada; Mood Disorders Research Unit, St Joseph’s Healthcare Hamilton, Hamilton, Ontario, Canada; Children’s Hospital of Eastern Ontario Research Institute, Ottawa, Ontario, Canada; Department of Psychiatry, University of Ottawa, Ottawa, Ontario, Canada; Assistant Clinical Professor, Department of Family Medicine, McMaster University, Hamilton, Ontario, Canada; Independent Researcher, Toronto, Ontario, Canada; Institute for Mental Health Policy Research, Centre for Addiction and Mental Health, Toronto, Ontario, Canada; Institute of Health Policy, Management and Evaluation, University of Toronto, Toronto, Ontario, Canada; Institute for Clinical Evaluative Sciences (ICES), Toronto, Ontario, Canada; Northern Ontario School of Medicine, Sudbury, Ontario, Canada; ICES North, Sudbury, Ontario, Canada; Mental Health and Addiction, Health Sciences North Research Institute, Sudbury, Ontario, Canada; Department of Psychiatry and Behavioural Neuroscience, McMaster University, Hamilton, Ontario, Canada; Department of Health Research, Evidence and Impact, McMaster University, Hamilton, Ontario, Canada; Biostatistics Unit, St Joseph’s Healthcare Hamilton, Hamilton, Ontario, Canada; Faculty of Health Sciences, University of Johannesburg, Johannesburg, South Africa; Department of Psychiatry, Queen’s University, Kingston, Ontario, Canada

**Keywords:** Cannabis, opioid disorders, perception, qualitative research, VAPE

## Abstract

**Background:**

Vaping is increasing in popularity. Vape products are offered in a wide variety and promise to reduce harms associated with cigarette smoking, among other claims. The motivations for vaping in patients with substance use disorder are largely unknown.

**Aims:**

To describe perceptions and motivations regarding vaping among patients with opioid use disorder (OUD) who vape.

**Method:**

A convergent mixed-methods study design was used, and individual, semi-structured interviews were conducted with 41 individuals with OUD who were receiving medication for OUD and also vaped. An inductive data-driven approach was employed to characterise perspectives on vaping.

**Results:**

The mean ages at which participants had been introduced to vaping and initiated regular vaping were 33.95 years (s.d. 12.70) and 34.85 years (s.d. 12.38), respectively. Daily vaping (85%) of nicotine, flavoured nicotine or cannabis was common, with 27% reporting vaping both nicotine and cannabis. Qualitative analysis identified 14 themes describing motivations for vaping, including viewing vaping as a smoking cessation tool, convenience and popularity among youth.

**Conclusions:**

Mixed-methods findings indicated that patients with OUD who vape perceived vaping to be healthier, cleaner and more convenient than cigarette and cannabis smoking, without appreciating the health risks. The perspectives reflected the importance of health education, guidelines and screening tools for vaping and could provide direction for healthcare providers and future vaping cessation programmes.

Vaping has gained popularity globally,^1^ with product offerings over the past several years having expanded vastly. New generations of vaporisers have departed from conventional models in both appearance and substance, probably owing to the explosion of new brands and evolving cannabis legislation. A review quantifying global trends in vaping found a lifetime prevalence of 23% and point prevalence of 11%, with both rates higher among men.^1^ Despite the expansion of product offerings, the impacts of vaping remain unknown. Some camps view vapes as harm reduction tools for smoking cessation, whereas others raise concerns over the inspiration of substance use in never-smokers, particularly in youth.^2^ The quick adoption of vaping has pre-dated any clear or credible consensus around its effects, which may explain the mixed messaging and unsubstantiated claims around its benefits. Indeed, some have claimed e-cigarettes to be less harmful than conventional cigarettes, before revoking such claims.^3^ Although safety remains unestablished, there is only modest confidence that nicotine e-cigarettes lead to better cessation outcomes than alternative cessation tools.^4^ Research has found more individuals substituting daily cigarettes for e-cigarettes, rather than alternative, tools approved by the Food and Drug Administration, with many transitioning to dual use rather than discontinuing cigarettes entirely.^5^

Despite the absence of trials, studies have found that vaping is significantly associated with the same harms and respiratory conditions as cigarette smoking, with long-term outcomes remaining poorly understood.^2^ Several cases of e-cigarette- and vaping-associated lung injuries have been reported,^2^ although their true incidence is obscured by limited vaping screening within primary care and emergency settings and, most recently, overlap with coronavirus 2019 (COVID-19) presentation.^6^ Patients with opioid use disorder (OUD) are at risk of vaping given their elevated risk for polysubstance use and health comorbidities; estimates suggest that more than 20% of patients on medication for OUD (MOUD) currently vape.^7^

## Vaping, OUD and COVID-19

The importance of studying the interplay of OUD, vaping and COVID-19,^8^ termed the ‘tripartite’ of epidemics, has been compounded by aggressive vaping marketing campaigns during the pandemic^9^ and rising fentanyl-related deaths.^8,10^ Recent work has shown the social and health implications of the COVID-19 pandemic for individuals with OUD.^11^ Studies have also found associations between vaping and COVID-19, including strong associations between COVID-19 diagnosis and past e-cigarette use in youth.^12^ In addition, aerosolisation in vaping has been shown to generate new compounds not present in the original solutions,^13^ and evidence of dose-dependent harms and extrapulmonary effects including neurodevelopmental effects is increasingly emerging.^14^ These findings together generate concern for the OUD population in treatment, as their exposure to opioids makes them vulnerable to respiratory illness, depression or toxicity, immunosuppression, and possible drug–drug interactions with evolving COVID-19 treatment.^15,16^ Socioeconomic challenges exacerbate these risks, specifically in the context of drug procurement activity and residential mobility.^17–21^

A lack of guidelines and screening tools precludes adequate surveillance of health effects of vaping and leaves patients at risk of vaping-related harms and vulnerable to social perceptions.^2,6^ Although the increasing prevalence of vaping has started to lead to a shift in perceptions of the safety of vaping in some,^22^ it is critical to understand perceptions of vaping in patients with OUD, whose health status, treatment, altered risk perception and decision-making, and decreased access to reputable information make them more susceptible to poor health outcomes with vaping.^15,19,23^ Current research has started to identify factors shaping vaping behaviour, including sensation-seeking, risk-taking behaviour and government policy regarding nicotine devices.^24,25^ However, surveys of vapers’ overall attitudes and perceptions of vaping in general non-clinical populations^24,25^ have shown a perception of low risk of vaping. Perspectives on vaping may differ between clinical (individuals with substance use disorder) and non-clinical (general population) groups. Investigation of population-specific factors may provide insight that could be leveraged to encourage quitting and public health policy.

## Rationale

Little is known about vaping behaviours or motivations in individuals with OUD, compared with other populations; this minimises the capacity for harm reduction, a core goal of MOUD treatment. Understanding patient perspectives is critical to adequate risk management.

## Objective

To explore perceptions of vaping among patients with OUD undergoing treatment in Ontario, Canada, using a convergent mixed-methods approach.

## Method

This study is reported according to the Consolidated Criteria for Reporting Qualitative Research^26^ (Supplementary Material 1, available at https://doi.org/10.1192/bjo.2025.6). The authors assert that all procedures contributing to this work comply with the ethical standards of the relevant national and institutional committees on human experimentation and with the Helsinki Declaration of 1975, as revised in 2013. All procedures were approved by the Hamilton Integrated Research Ethics Board (no. 12602).

### Study design

**Fig. 1:**
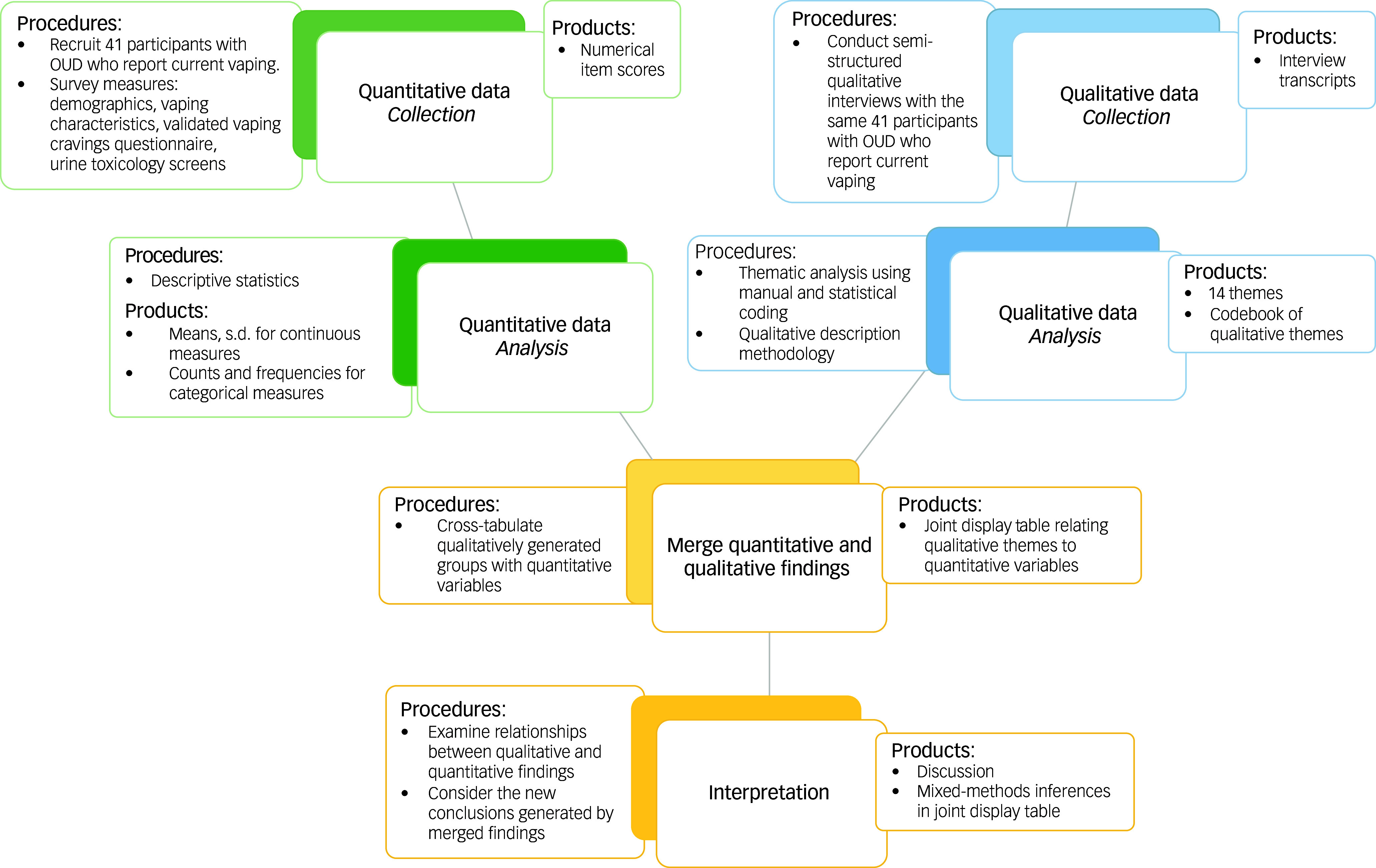
Summary of quantitative and qualitative data collection, analysis and integration. OUD, opioid use disorder.

Semi-structured, open-ended interviews (Supplementary Material 2, Interview Guide) were conducted with individual OUD patients. A convergent mixed-methods approach was used; qualitative and quantitative data were compared and integrated to enable thorough understanding of the research problem. Collection, analysis and integration of data are summarised in Fig. 1. The virtual format was selected to minimise risk and COVID-19 related disruptions. The qualitative component was underpinned by qualitative description methodology.

The aim of the study was to identify and describe perceptions about and motivations for vaping among individuals on MOUD who vaped and were enrolled in community-based addiction clinics in Ontario, Canada. An inductive thematic analysis with a data-driven approach was used to yield ‘straight’ descriptions of themes related to the phenomenon of interest most appropriate for the selected study design. We engaged with an individual with lived experience to ensure comprehension of study materials and/or questions and their relevance to participants’ lived experience (Supplementary Material 6: Patient and Public Involvement).

### Participant selection

Participants were 16 years of age or older, had an OUD diagnosis (DSM-5)^27^ for which they were receiving active treatment in community addiction clinics. Diagnosis was confirmed against clinic electronic medical records, with no restrictions on phase of disorder. Patients lived in Ontario, spoke English and currently vaped.

### Recruitment and setting

**Fig. 2:**
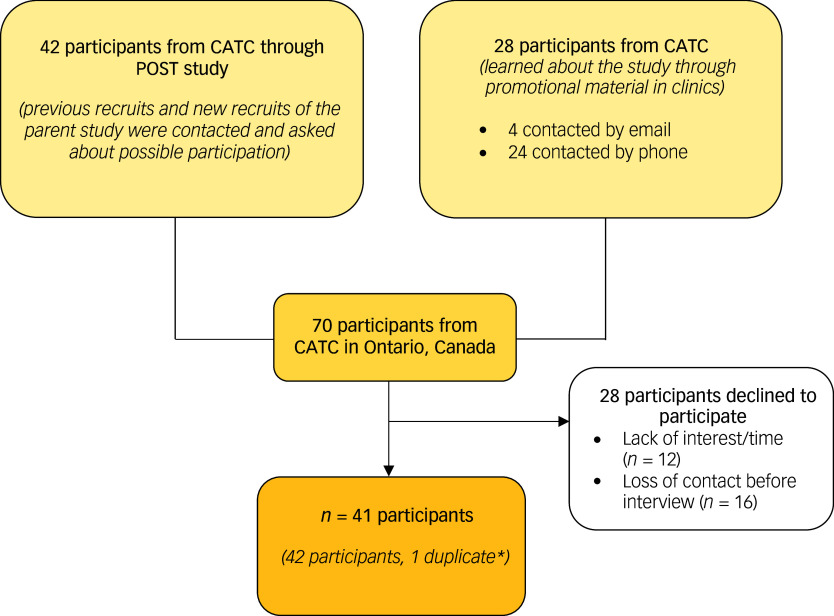
Participant flow diagram. The study team discussed possible participation with 70 participants in total. Participants were recruited using promotional material for the VAPE study (*n* = 28) or were contacted by the study team after participating in the POST (Pharmacogenetics of Opioid Substitution Treatment) study, the parent cohort study. In all, 41 participants were enrolled; 33 enrolled participants participated in both the POST and VAPE studies. *A duplicate respondent who provided a false name during their second participation; data from the first interview were used for analysis. CATC, Canadian Addiction Treatment Centres.

Remote, purposeful recruitment was conducted per the study protocol (Supplementary Material 3). Approval was obtained from the Hamilton Integrated Research Ethics Board to contact via phone new participants from the clinics and previously enrolled eligible participants from the ongoing parent cohort study.^7^ Participants were recruited from February 2021 to April 2022. Seventy individuals were approached, with 41 unique individuals consenting to participate (Fig. 2). Verbal consent was obtained from all participants and audio recorded; where possible, additional written consent was obtained. Reasons for non-participation were lack of interest (*n* = 12) and loss of contact before the interview (*n* = 16). Participants received a CAD$10 gift card for participating.

The target sample size was 50, the upper limit of the range recommended for qualitative studies.^28^ Recruitment was prespecified to terminate when saturation was reached; this occurred at 41 participants, when there was agreement within the team that continued enrolment was unlikely to generate new themes.

As interviews were completed by phone, the setting of participation differed based on the participant’s choice. Participants were offered phone access and a private space in the clinic (where they receive treatment) or selected their own time and location if they had a personal phone to use. Owing to the virtual format, it is possible that the setting or the presence of non-participants in the surroundings may have affected participants’ responses.

### Research team characteristics

The interviews were performed by A.D., B.P. and N.S., senior female graduate students who were enrolled in or had completed PhD-level studies. All interviewers had completed qualitative interview training, had previous experience from prior studies and had completed practice interviews. No professional or therapeutic relationships existed between interviewers and participants. During the consent procedure, participants were told the research goal was to better understand their personal experience with vaping.

### Data collection and analysis

#### Qualitative data collection and analysis

A qualitative interview guide (Supplementary Material 2) was developed in partnership with a qualitative interview expert to generate discussion around participants’ experiences, using open-ended questions. The guide was constructed and then discussed among the research team. An individual with lived experience was consulted to provide input on content, structure and phrasing of the qualitative interview and data analysis. The use of telephone interviews precluded data generation from visual cues; therefore, interview transcripts from audio recordings were the sole source of data for coding and analysis. The research team did not plan for repeat or correction of interviews with participants owing to demonstrated infeasibility of follow-up with this population.

The transcript data were coded independently by two authors (A.D. and B.P.) and then together to discuss initial patterns and themes. Any disagreements on transcription or content were discussed between at least two interviewers (A.D., B.P., N.S.) to reach a consensus. Manual, partially coded data were analysed using NVivo 12 Qualitative Data Analysis Software (QSR International).^29^ Text searches were conducted; then, word frequency queries were generated with the terms, arranging information according to frequency. Manual qualitative analysis and initial patterns identified through word count queries were then analysed together to refine and validate themes and codes. Results were shared with the research team for feedback, including experts in opioid addiction and mental health (N.S. and Z.S.), supporting the description of themes.

#### Quantitative data analysis

During interviews, demographic and vaping characteristics were collected. Self-reported clinical characteristics including type of MOUD treatment, current MOUD dosage, current cigarette use, average daily cigarette consumption and body mass index were collected. Urine toxicology screens (UTSs) from medical records obtained using FaStep Assay were used as an objective measure of drug use (Trimedic Supply Network Ltd, Concord, Ontario, Canada).^30^ UTSs were ordered as clinically indicated by the participant’s treating physician.

Vaping behaviours were explored by collecting the following characteristics: age when first introduced to vaping, age at which vaping regularly was initiated and number of years vaping. Participants were also asked to classify their vaping frequency as ‘everyday’, ‘every other day’, ‘2–3 times per week’, ‘once per week’ or ‘2–3 times per month.’ The average amount of money spent on vaping per week was reported in Canadian dollars. Data on substances vaped were extracted from free-text responses, in which participants were asked to report all the substances that they regularly vaped (defined as at least twice per month). Participants were asked to report the most common setting for vaping, with response options of ‘alone’, ‘with one other person’, ‘with two or three other people’ and ‘with four or five other people.’ Participants were asked to report reasons for vaping.

For demographic, clinical and vaping characteristics, means and standard deviations are presented for continuous variables, and counts and percentages are provided for categorical variables. UTS data are presented as the percentage of positive screens (the number of positive screens divided by the number of total screens performed, multiplied by 100%) for each drug. As several individuals reported use of more than one illicit substance, counts may exceed the total sample size.

#### Convergent mixed-methods analysis

A convergent analysis approach was used to integrate and assess agreement between qualitative and quantitative data. For each qualitative theme, a demographic description of the participants contributing to that theme is provided, alongside analysis of related, complementary quantitative variables.

## Results

### Sample description

Participants were mostly female patients with OUD (58.5%), of European descent (78%), on methadone treatment (85.4%; mean dose 84.36 mg/day, s.d. 48.76), with a few on buprenorphine (14.6%; mean dose 17.83 mg/day, s.d. 8.64). Demographic and vaping characteristics are provided in Tables 1 and 2, respectively.

**Table 1:** Demographic characteristics

Characteristics	*n* = 41
Age in years; mean (s.d.)	39.54 (37.29)
Females; *n* (%)	24 (58.5)
Males; *n* (%)	17 (41.4)
Cisgender female; *n* (%)	23 (56.1)
Cisgender male; *n* (%)	17 (41.4)
Nonbinary; n (%)	1 (2.4)
Ethnicity; *n* (%)	
European	32 (78.0)
Native North American	3 (7.3)
Mixed	5 (12.2)
Other	1 (2.4)
Marital; *n* (%)	
Single, never married	24 (58.5)
Married	3 (7.3)
Common law or living with a partner	7 (17.0)
Widowed, separated or divorced	7 (17.0)
Education; *n* (%)	
Grade 1–8	7 (17.0)
Grade 9–12	22 (52.7)
College/University/Master’s/PhD	11 (26.8)
Other (trade school or none)	1 (2.4)
Methadone MOUD; *n* (%)	35 (85.4)
Buprenorphine MOUD; *n* (%)	6 (14.6)
Current methadone MOUD dose in mg; mean (s.d.)	84.36 (48.76)
Current buprenorphine MOUD dose in mg; mean (s.d.)	17.83 (8.64)
Age in years when first introduced to vaping; mean (s.d.)	33.95 (12.70)
Age in years when initiating vaping regularly; mean (s.d.)	34.85 (12.38)
Number of years vaping; mean (s.d.)	4.58 (4.31)
Current cigarette use; *n* (%)	27 (65.9)
Cigarettes per day; mean (s.d.)	10.65 (6.11)
Body mass index; mean (s.d.)	30.67 (35.87)
Comorbid conditions; *n* (%)	
Autoimmune	3 (7.3)
Cardiovascular	5 (12.2)
Gastrointestinal	2 (4.9)
Respiratory	5 (12.2)
Mental health (anxiety, mood disorders, stress disorders)	21 (51.2)

MOUD, medication for opioid use disorder.

**Table 2:** **Vaping characteristics,** ***n*** **= 41**

Vaping frequency, *n* (%)	
Every day	35 (85.4)
Every other day	2 (4.9)
2–3 times per week	3 (7.3)
2–3 times per month	1 (2.4)
Dollars per spent on vaping per week (CAD/week); mean (s.d.), *n* = 38	25.41 (29.32)
Range in dollars spent on vaping per week (CAD)	0–100
Substances vaped, *n* (%)^a^	
No. who report vaping nicotine	26 (63.4)
No. who report vaping flavoured nicotine	13 (31.7)
No. who report vaping water flavour (no nicotine)	1 (2.4)
No. who report vaping cannabis (THC and/or CBD)	18 (43.9)
No. who report vaping dual use of nicotine and THC and/or CBD	11 (26.8)
Most common setting for vaping; *n* (%)	
Alone	35 (85.4)
With one person	3 (7.3)
With two or three people	2 (4.9)
Other (equally with others and alone)	1 (2.4)
Reasons for vaping; *n* (%)	
To get high	13 (31.7)
Calmness and/or relaxation	34 (82.9)
Others around me are using it	9 (23.1)
For pleasure	21 (53.8)
Stress relief	30 (76.9)
Boredom	22 (56.4)
Social anxiety relief	19 (48.7)
Use a vape instead of other substances	29 (74.4)
Substance use withdrawal or relief of craving symptoms	32 (82.1)
Craving or withdrawal relief from opioids (i.e. fentanyl)	8 (20.5)
Craving or withdrawal relief from cigarettes (i.e. nicotine, tobacco)	24 (61.5)
Craving or withdrawal relief from cocaine (i.e. crack, cocaine)	5 (12.8)
Craving or withdrawal relief from marijuana	3 (7.7)
Craving or withdrawal relief from methadone	2 (5.1)
Craving or withdrawal relief from methamphetamine	1 (2.6)
Urine toxicology screens (reported as percentage positive; positive screens/total screens)	
Opioids	16.2% (6/37)
Benzodiazepines	16.2% (6/37)
Amphetamines	27.3% (3/11)
Cannabis	33.3% (1/3)
Cocaine	24.2% (8/33)
Methamphetamine	50% (2/4)
Questionnaire of Vaping Cravings score; mean (s.d.)	36.18 (15.88)

CAD, Canadian dollars; CBD: cannabidiol; THC, tetrahydrocannabinol.

a. As participants were able to report more than one substance, the number of substances exceeds 100%.

### Qualitative results

Manual analysis of qualitative interviews yielded 14 themes, and NVivo statistical analysis yielded 12; comparison of analyses strengthened the identified themes and codes, and all 14 themes were explored to ensure inclusivity and more comprehensive reporting of nuances. Table 3 shows all 14 themes; integrations with corresponding quantitative data are visualised in a joint display table (Supplementary Material 4). Figure 1 summarises the integration of results. Supplementary Material 5 contains the qualitative codebook.

**Table 3:** Fourteen qualitative, data-derived themes relating to perceptions and motivations for vaping

Theme	Examples
Perceived convenience	‘Um. cause… cause like I buy a cart– like I’ll buy a cartridge and then I’ll buy a pack of cigarettes, and if I don’t have the money, then I just use the vape.’ (participant 1; female, 36) ‘And sometimes it’s just having a couple of inhales of […] the vaporiser is quicker than trying to smoke a half cigarette.’ (participant 5; male, 25)
Perceived personal benefit (non-health related)	‘Yeah. Like, my hands didn’t smell afterwards. And stuff…’ (participant 1; female, 36) ‘Sometimes it helps with relaxation when I’m at home and I don’t wanna move, and I can just relax and I have my e-cigarette with me and uh, veg on the couch.’ (participant 3; female, 33)
Perceived agency and/or independence	‘Yeah. So like, I mean, I just like the ability to, um, begin, and and stop whenever I choose.’ (participant 4; female, 41) ‘Um, I really, don’t do much research into that kinda stuff, like I just kinda go with my day and go with the flow, and um, for me, not to have to smoke a full cigarette or not to – like I just find that it, it fixes my craving, um, within one puff. Which, as I say, one puff is like 5 times a day, that’s like a cigarette in a day.’ (participant 4; female, 41)
Perceived smoking reduction	‘I’m more addicted to cigarettes, like I’ve smoked cigarettes since I was 14 years old. And with vaping […] I usually just do it to try and see if I can go a day or – a couple days without smoking cigarettes.’ (participant 5; male, 25) ‘It was usually a matter of going back to cigarettes, or going down on the cigarettes and choosing the vape more.’ (participant 11; male, 43)
Vaping is perceived to be a smoking cessation tool	‘So, um, like I’d say the first month I was still smoking, like cigarettes, strongly. But after that, and I, um, I almost went three months straight without, no cigarettes.’ (participant 7; male, 33) ‘Um, yeah because I had heard of it as an alternative to cigarettes. It was all because I wanted to quit cigarettes so I was looking for options, and there hasn’t ever been many options other than like, you know, nicotine gum and stuff, and that stuff just doesn’t work.’ (participant 9; male, 31)
Perceived changes in substance use	‘Uh, the one reason is because I had been trying to get off of the fentanyl. And, if I couldn’t get it, it would take away the physical ummm, withdrawal symptoms that I was – uh – going through…’ (participant 29; female, 53) ‘Not at all, it’s not a social thing at all right now, it’s more so, yeah like boredom stress release, especially since I quit smoking other things which were kinda my coping mechanism.’ (participant 14; female, 18)
Perceived lack of information and/or understanding of vaping	‘Um, I am wary of vaping because there’s not that much – I’m not sure about the statistics on it? I know cigarettes cause cancer and stuff like that, but, I feel like vaping has gotta be – there’s gotta be some, some sort of medical downfall to vaping. Um, it’s just another toxin that we’re putting into our bodies.’ (participant 3; female, 33) ‘Um, I just, a question mark on negative health effects [unintelligible], but uh, I haven’t done too much research into it myself so.’ (participant 13; male, 36)
Perceived social benefits motivate vaping behaviours	‘Um, he doesn’t like me smoking so he suggested I started to vape.’ (participant 14; female, 18) ‘. . . I – I like the way it doesn’t make my clothes smell, it doesn’t make my hair smell. Um, it looks a lot more friendlier around my kids.’ (participant 24; female, 33) ‘It’s a little cleaner. Um, in, in society now, it seems to be more accepted.’ (participant 37; female, 52)
Perceived positive health effects of vaping	‘It [coughing/breathing] got hugely better, cause like I’m not smoking a half and a pack a day.’ (participant 6; female, 29) ‘So, when I realised that I could get my daily THCs [tetrahydrocannabinol] for cheaper and easier and less… health – like – harm reduction wise.’ (participant 8; male, 36)
Perceived negative health effects of vaping	‘I coughed and uh didn’t care for it very much.’ (participant 15; female, 59) ‘Yes, I do get short of breath when I vape.’ (participant 15; female, 59) ‘I found at first, it uh, if I was smoking it too much, I felt nauseous, and I would get headaches.’ (participant 6; female, 29)
No perceived impact of vaping on MOUD	‘Uh, no. I, uh – if it does, I haven’t noticed it…’ (participant 5; male, 25) ‘N-n-no, no, no, no interaction. It’s a totally – apples and oranges. For uh – […] Or fruits and vegetables, no-no-no, no bearing, one doesn’t have any bearing on the other.’ (participant 19; male, 64)
Vaping has some effects on MOUD	‘I find that it makes the methadone seem to last longer.’ (participant 29; female, 53) ‘…a lot of people find the same thing as me that it’s really complimentary. And um, weed fills in the cracks where methadone is not perfect cause no medication can be perfect, right?’ (participant 36; nonbinary, 24)
Perception that vaping is for the youth	‘Like a little younger, youths, and even uh, students, and I find, uh, their, uh, like a lot of younger people are using it rather than older people, right?’ (participant 5; male, 25) ‘The only thing I’ve heard is that the young kids, the teenagers, they, they get into the, they vape around with no nicotine in it, or something, I don’t know.’ (participant 12; female, 46)
Vaping to get high	‘Well I felt that I could control my high more through vaping.’ (participant 34; female, 69) ‘So, I’ll just vape it, like just to get high. And it will be like a hit here and there.’ (participant 7; male, 33)

MOUD, medication for opioid use disorder.

This study collected perspectives from 41 individuals on MOUD treatment who vaped. The mean interview length was 8 min. The main themes of this analysis were perceived personal benefits of vaping, use of vaping to support smoking reduction or cessation, and lack of knowledge and understanding of vaping. Below, we describe each qualitative theme alongside corresponding quantitative results.

#### Perceived convenience; perceived personal benefit, non-health related; perceived agency and/or independence

Personal benefits, including cost, were found to be important among nicotine and cannabis vapers. Participants found that vaping allowed them to spend less on cigarettes, through reduction or cessation. Participants highlighted the convenience of vaping, suggesting that it is easier and cleaner and positively associated with enjoyment and comfort owing to ease of consumption and lack of smell. Many participants’ reported vaping ‘for pleasure’, and participants generally disagreed with the statement ‘vaping would make me feel happier now’.

Vaping was associated with agency and independence. Participants reported feeling more in control of their consumption (and therefore time) than with cigarettes, which afforded them independence and more freedom to do other activities.

#### Perceived smoking reduction; vaping is perceived to be a smoking cessation tool

Participants viewed vaping as a smoking cessation tool, with some regarding it as harmful and listing negative effects. Vaping was described as being driven by desires to quit or reduce cigarette smoking, eventually becoming an alternative or sometimes supplementary means to consumption. Concurrent smoking was common (67%, mean 10 cigarettes daily); there was little difference in daily consumption between those who reported being able to reduce consumption and those who did not (10 *v*. 11 cigarettes/day).

#### Perceived changes in substance use; vaping to get high

Participants perceived vaping to be conducive to abstinence from drugs and reported that it helped to curb cravings (81%) or served as a substitute (74%) for illicit drugs. Others reported vaping to ‘get high’. UTSs showed positivity for methamphetamine (50%), cannabis (33.3%), amphetamines (27.3%), cocaine (24.2%), opioids (16.2%) and benzodiazepines (16.2%).

#### Perceived lack of information and/or understanding of vaping

Overwhelmingly, patients reported lack of knowledge on the health impacts of vaping and generally did not report active interest in or urgency regarding personal research to learn more. ‘Um, I just, a question mark on negative health effects […] I haven’t done too much research into it myself’ (participant 13; male, 36).

#### Perceived social benefits motivate vaping behaviours

Vaping appeared to have a social component, with participants describing initiation through social settings and positive social interactions; this corresponded to our quantitative data, in which 20% of participants reported vaping because others around them were and 15% reporting that they typically vape around others.

#### Perception that vaping is for the youth

Vaping was perceived as associated with youth in general and not specific to the OUD population, with participants reporting the belief that the flavours available attract youth.

#### No perceived impact of vaping on MOUD; vaping has some effects on MOUD

Perceptions were divided on the effects of vaping on MOUD treatment. UTS data for opioids showed 16% positivity. Those reporting positive effects of vaping on MOUD treatment tended to vape cannabis (8/10). Although this suggests an impact of cannabis on MOUD, comparison of UTSs in cannabis vapers and non-cannabis vapers showed higher positivity for opioids and other drugs in those who vaped cannabis (20% in cannabis vapers versus 13.6% in nicotine or water-flavour only).

#### Perceived positive health effects of vaping; perceived negative health effects of vaping

Participants perceived vaping to be associated with benefits with respect to stress and anxiety and improved respiratory symptoms. Yet, more than half reported mental health concerns, including mood disorders. Perceived positive physical effects of vaping were often mentioned yet rarely discussed independently of cigarettes. A few participants perceived vaping to be associated with negative respiratory symptoms, with most patients reporting that symptoms eased with adjustments to strength or inhalation. Few mentioned harms or the addictive potential of vaping; desires to quit were rare.

## Discussion

This study aimed to explore themes of reasons for vaping and perceptions of use among patients in treatment for OUD. The main finding of this study was that participants felt vaping was critical to their smoking cessation goals and driven by fewer perceived harms and more personal benefits. Participants had little knowledge about vaping and limited awareness of its health effects. Finally, the findings suggested that patients with OUD view ‘vaping’ a substance as inherently different from other methods of administration. Our findings were congruent with research in patients with substance use disorders and in the general population. We built upon previous work and identified additional study-population-specific motivators and perceptions driving vaping.

Qualitative analyses showed that patients with OUD had initiated vaping to support smoking reduction and cessation, echoing other research in patients with substance use disorders.^31,32^ Our findings align with previous research showing that individuals initiate vaping to ‘substitute’ cigarettes.^32,33^ Although patients with OUD in this study were motivated to vape to reduce smoking, we found that smoking remained common (67%), with similar daily smoking in those who did and did not vape (10 cigarettes/day in vapers *v*. 14–15 cigarettes/day in non-vapers).^7^ Individuals in the study also used vaping for approximately 4.5 years. The concurrent smoking and vaping over several years questions the effectiveness of vaping as a smoking cessation aid as perceived by the study participants.

Although this study did not address whether vaping led to greater total consumption of cannabis or nicotine, our findings showed everyday dual use of smoking and vaping, which may pose additional health risks. A study comparing dual users (vaping and smoking), cigarette-only smokers and never cigarette smokers found that those who smoked and vaped had greater nicotine dependence and nicotine metabolite levels than cigarette-only smokers.^34^ Metabolite levels also differed based on nature of dual use when comparing everyday smokers with everyday vapers, someday smokers with everyday vapers, and everyday smokers with someday vapers.^34^ Our findings support concerns about greater nicotine consumption, nicotine tolerance and poly-tobacco use. We also found that those who vaped have little understanding of the health effects of vaping.^24^ This is concerning, because observations indicate trends of increased vaping over the past several years, despite mounting evidence of its harmful effects (i.e. e-cigarette- and vaping-associated lung injuries) and a lack of evidence that vaping can support individuals with smoking cessation.^4,8^

Most study participants reported positive perceptions of vaping compared with cigarettes, including positive health benefits and social benefits, in addition to an assumed role of vaping in smoking cessation or reduction. Most participants conveyed feelings that vaping was less addictive than smoking. Participants reported vaping being healthier than cigarettes and showed ‘dedication’ to vaping.^32,33^ They felt that nicotine consumption through vaping was safer compared with smoking cigarettes, suggesting differences in methods of consumption of nicotine to be associated with different health effects despite still consuming nicotine. Positive health effects were reported more often than negative health effects, and few participants mentioned intent to quit vaping, implying that perceived or actual harms were not important enough to change behaviours. Neutral responses to the statement ‘I am missing vaping right now’ and moderate craving scores were aligned with divided perspectives on vaping being addictive, although prevalent daily vaping suggested possible dependence.

It is important to consider whether the perception of vaping in this population may be a function of comparison with other substances. For example, effects of vaping may be underreported or viewed as less important because of the more immediate and serious risks of opioid use (overdose, death). Differential risk perception in patients with OUD is supported by evidence showing that smokers with substance use disorders view smoking as less serious than use of other substances, and that there is low risk perception regarding vapes in young vapers.^24,35^ This also aligns with reports of negative associations between poly-tobacco use and perceived harm.^36^ Concordantly, our results show that individuals with OUD view vaping as inherently different from smoking with respect to risk, health effects and purpose for use. It is likely that vaping is seen as even less serious than other methods of consumption of substances, which may explain lower attentiveness to or awareness of vaping-related adverse effects and continued vaping within this sample.

Perceived pleasure, convenience, ease of use and savings were found to be drivers of vaping behaviour in individuals with OUD. Although these drivers of behaviour may be applicable to the general population, they are likely to be especially important in individuals with OUD, who are known to experience social and economic challenges, as well as difficulties with impulsivity and control over substance use.^21,23^

Vaping was perceived to have a positive impact on OUD treatment with respect to interaction with MOUD and reductions in illicit substance use. Positive effects on MOUD were more common to cannabis vapers, in whom it was perceived to strengthen the therapeutic benefit and duration effect of methadone and improve treatment adherence. These perceived effects may result from psychoactive properties of cannabis, which may modulate cravings or produce analgesic effects. Participants believed they had lower cravings and reduced their use of or ceased using drugs such as crystal methamphetamine and opioids; however, this contradicted quantitative data that showed continued use of these substances. Urine drug screens for individuals reporting ‘using vaping to abstain from drugs’ showed near equivalent positivity for illicit drugs to that of those who did not report that as a reason, raising further questions about vaping as tool for abstaining from illicit drugs. Similarly, current evidence suggests that past-month cannabis use is not associated with more or less opioid use in OUD patients on treatment;^37^ there is mixed evidence regarding positive effects of tobacco on coping with urges for other drugs, with some studies suggesting perceived modulation of opioid cravings.^35,38^ With ‘getting high’ mentioned as a reason for vaping, together with the persistent illicit drug use in this study, we suggest that vaping may be perceived to help individuals to manage illicit drug cravings while not actually producing cessation. There is a need for research on the effects of vaping and cannabis on MOUD and drug use.

Many perceived vaping to be associated with young age, a belief probably emerging from greater media coverage around youth vaping and regulatory changes to flavours to curb use in young adults. However, the mean age of this sample (40 years) aligned with the average age of patients on MOUD;^7^ the mean ages for trying vaping and regularly vaping within this sample were 34 and 35 years, respectively. This suggests that vaping is also common in adults with OUD. Although flavours are believed to appeal to younger individuals, this was also common among older participants (>30 years).

Similar to smoking, vaping initiation commonly emerges in social spaces; many indicated the presence of others as a reason for vaping. Our findings are congruent with previous work showing associations of smoking with social identity and peer influence and match social associations with vaping reported elsewhere.^24^ Like smoking, vaping may be linked to identity and belonging, with possible effects on vaping patterns.^39^

### Integration and interpretation of findings

Our findings suggest that in the absence of clear direction or accessible, definitive evidence on vaping, patients may rely on personal and social perceptions of vaping, especially those that stem from actualised experiences of perceived pleasure and convenience (i.e. ease of use and cost). Lack of knowledge may leave patients vulnerable to bias, making decisions about vaping on the basis of directional research and personal experiences (confirmation and social biases). Decision-making aids and patient-friendly materials are needed to provide information on the risks of vaping, given the strong motivators shaping behaviour. Research and knowledge translation must prioritise discussion of vaping-specific concerns, such as harms related to vaping devices and aerosolisation.^13^ With new devices and new materials coming to market, users must remain vigilant when selecting brands, products and substances; guidelines must empower low-risk product selection.^13^

Vaping appeals to patients with OUD owing to the removal of barriers such as smell, taste and cost, as well as the introduction of flavours delivered through a convenient, ‘futuristic’ product design. Coupled with pervasive perceptions that ‘vaping is healthier than cigarettes’ and the largely uncharacterised risks of vaping devices, our results suggest that vaping may beget continued substance use and intrigue those previously deterred by the unpleasant effects of smoking. Without adequate awareness, individuals who vape to stop smoking may continue to vape. Educational programming must dispel misconceptions that vaping is nonaddictive or free of substance-related effects.

Vaping appears to be a convenient means of soliciting and controlling one’s ‘high’ and may become an entry point through which individuals initiate or remain within cycles of substance use. As cannabis is often perceived as ‘less harmful’ than illicit substances and legalisation becomes more prevalent, it is critical to better understand and communicate the risks of vaping cannabis. Understanding vaping within this population is critical to improving overall health outcomes and preventing dual use of vapes and cigarettes. As individuals with OUD experience are at risk of comorbid conditions requiring significant time and intensive healthcare utilisation, population-informed strategies for prevention of continued or additional substance use must be generated and implemented.

### Limitations

The qualitative descriptive design used within this study was data-driven and lacked the formality of alternative, theory-based approaches. The short interview duration may have affected the depth of the data presented. The findings must be understood considering lifetime smoking. As treatment for OUD is most common among adults (>16 years old), perspectives captured here may be biased to exclude younger youths with OUD. The results are likely to have been shaped by participation, social desirability and recall bias.

### Implications and future research

Future research should study the health effects associated with vaping, particularly within individuals without a history of smoking to separate out vaping effects. Health records may be used in future to provide objective measures of health and resource utilisation related to vaping, with use of non-vaping comparator groups. The present study shows the perspectives of a population at risk of worsening health, whose reasons for vaping may be applicable to other patients with addiction on treatment within a publicly funded healthcare environment. Largely, the results of this study suggest that intentions for vaping do not correspond to changes in smoking or substance use behaviour. Vaping is viewed as distinct from smoking, despite continued consumption of a particular substance, and individuals with OUD lack understanding of the health impacts of vaping. Thus, this work questions whether vaping supports smoking cessation, or whether it enables easier and cheaper consumption, using devices for which sufficient risk assessment has yet to reach consensus.

This work also contributes to the ongoing study of patterns of vaping; understanding of these patterns is critical to developing guidelines for clinical decision-making, given the lack of patient knowledge and the lack of screening for vaping in healthcare setting, and the lack of it affects the impact of conversations between patients and healthcare providers. Harnessing population-specific motivators could provide direction for more effective cessation initiatives, as well as potentially supporting promotion of approved cessation-methods and informing education on the realities of vaping.

## Supporting information

D’Elia et al. supplementary material 1 D’Elia et al. supplementary material

D’Elia et al. supplementary material 2 D’Elia et al. supplementary material

D’Elia et al. supplementary material 3 D’Elia et al. supplementary material

D’Elia et al. supplementary material 4 D’Elia et al. supplementary material

D’Elia et al. supplementary material 5 D’Elia et al. supplementary material

D’Elia et al. supplementary material 6 D’Elia et al. supplementary material

D’Elia et al. supplementary material 7 D’Elia et al. supplementary material

D’Elia et al. supplementary material 8 D’Elia et al. supplementary material

## Data Availability

The data for this study can be made available upon reasonable request.
