# Supplementary material for: Effects of vaping on physical and mental health in at-risk populations (VAPE): mixed-methods study of motivations for and perspectives on vaping in patients with opioid use disorder
Source: BJPsych Open. 2025 Apr 2;11(3):e75. doi: 10.1192/bjo.2025.6 (PMC12052573; doi:10.1192/bjo.2025.6)
Supplement: D’Elia et al. supplementary material 1 — D’Elia et al. supplementary material [file S2056472425000067sup001.docx]

**Appendix F: Patient and Public Involvement**

Patient and public involvement (PPI) was a component of this study through the engagement of an Individual with Lived Experience (IWLE) with vaping. This individual was involved in order to inform various study components to ensure the following: comprehension of the participant population, and relevance and congruity of study materials/questions with lived experience.

*Engagement of IWLE*

An IWLE with vaping was engaged throughout the following phases of the research process: funding acquisition, study design, development of qualitative and quantitative interview questions, and interpretation of study findings; contribution to knowledge translation will be sought from the same IWLE. Input was used to develop study questions, support robust data collection, and inform selection of outcome measurements (i.e., questions about frequency of use, etc). Involvement at this phase ensured participant comprehension of study materials and questions by incorporating correct, commonly used terminology. The IWLE was involved in interpretation of mixed-methods findings to improve the rigour of study methodology, and to ensure the nuance of study conclusions by capturing IWLE perspectives. Ongoing meetings with senior graduate students facilitated IWLE involvement at each phase. Initial versions of the manuscript were shared with the IWLE to collect feedback and contribute to edits of lay sections for inclusion in the final manuscript; the IWLE is listed as a coauthor.

*Contribution of IWLE to the study*

IWLE had critical contributions to the study design and study questions; IWLE were not involved in participant recruitment. Involvement was successful likely because the research team was experienced at including IWLE in research projects. Participation from the onset of the study (i.e., funding acquisition and study design) likely ensured the fluency of IWLE with the goals of the research project and led to meaningful input throughout data collection and analysis phases.

*Key Lessons*

Discussion of study goals and early establishment of working relationships between the IWLE and specific team members facilitated the comfort of the patient partner. These steps created a safe environment for perspective sharing that positively impacted IWLE contributions to the study, and should be repeated in future work. PPI influence was limited due to partnership with only one IWLE; this limitation could be overcome in future studies by engaging multiple patient partners.

The involvement of PPI within this study was critical for ensuring the quality of the study data through checking the alignment of study questions and materials with the perspectives and understanding of a lay audience. The IWLE lacked fluency with traditional research models and standards which impacted the extent to which IWLE could shape study methodology. Additional education and training of IWLE may empower contributions in future research with PPI.
