# Supplementary material for: Effects of vaping on physical and mental health in at-risk populations (VAPE): mixed-methods study of motivations for and perspectives on vaping in patients with opioid use disorder
Source: BJPsych Open. 2025 Apr 2;11(3):e75. doi: 10.1192/bjo.2025.6 (PMC12052573; doi:10.1192/bjo.2025.6)
Supplement: D’Elia et al. supplementary material 4 — D’Elia et al. supplementary material [file S2056472425000067sup004.docx]

**Appendix C: Qualitative Interview Guide**

As you know, we are doing this interview in the hopes of getting your perspective on vaping as a whole along with some questions about vaping while receiving medication assisted treatment for opioid use disorder. 

1. How were you first introduced to vaping? 
2. Can you tell me a bit about your experience of vaping? 
3. What do you vape? 
4. Why do you vape? 
5. Have you ever tried to quit vaping? Why did you try to quit? What was quitting like for you? Or What is it about vaping that leads you to continue with it?
6. Do you find vaping to have any effect (positive or negative) on your physical health (e.g. EVALI, COVID-19, smoking cessation tool)? 
7. Do you find vaping to have any effect (positive or negative) on your medication assisted treatment? 
8. Is there anything else that you would like to tell me about your experience with vaping that you think is important for me to know?
