# Supplementary material for: Effects of vaping on physical and mental health in at-risk populations (VAPE): mixed-methods study of motivations for and perspectives on vaping in patients with opioid use disorder
Source: BJPsych Open. 2025 Apr 2;11(3):e75. doi: 10.1192/bjo.2025.6 (PMC12052573; doi:10.1192/bjo.2025.6)
Supplement: D’Elia et al. supplementary material 5 — D’Elia et al. supplementary material [file S2056472425000067sup005.docx]

**Appendix D: Joint Display Table of Qualitative and Quantitative Findings**

| Major Domains | Quantitative measures | Qualitative findings | | Mixed-methods inferences |
| --- | --- | --- | --- | --- |
|  |  | Sample description | Qualitative subcategories |  |
| Personal benefits | **Average amount spent per week (CAD/week)**  Mean $25.41 per week (SD 29.32) | N= 20  10 female, 10 male  **Substances vaped:**  Cannabis only, n=5  Nicotine & Cannabis, n=6  Nicotine only, n=9 | Cost   - Comments discussing or initiating because it is cheaper or because others have recommended it as a method for cost savings.   Autonomy   - Comments related to regaining independence over the time spent in acquiring nicotine and managing cravings. | *Congruence*   - Participants perceived vaping to help with cost and agency, reported among both those vaping cannabis and nicotine. - Quantitative reports of the average amount spent on vaping products per week is over $25 CAD. - Considering the average cost of a 25 pack of cigarettes in Ontario ($14 CAD), at a mean of 10 cigarettes per day reported by this sample at the time of interview, weekly cost would be approximately $39.20. - It appears that the cost of purchasing cigarettes is much greater than vaping products, suggesting that vaping is permitting cost-savings, corresponding with participant perceptions. |
| Personal Benefits | **QVC item: “Vaping would make me feel happier now” (on a scale of 1 to 7, which denote strongly disagree and strongly agree, respectively, and 4 denotes “neither agree nor disagree).**  Range 1-7  Mean response = 3.48. | n=27  16 female, 11 male  **Substances vaped:**  Cannabis only, n=5  Nicotine only, n=13  Nicotine & Cannabis, n=8  Water-flavour only, n=1 | Enjoyment   - Comments pertaining to enjoying vaping due to qualities such as flavour, and responses stating how the flavours of vaping were the only thing preventing them from using cigarettes again. | *Expansion*   - Participants descriptions of enjoyment and pleasure resulting from vaping disagree with the average ratings of disagreement or neutrality toward the idea that “vaping would make them feel happier”. - This suggests that motivations to vape involve seeking pleasure, and may be unrelated to happiness. |
| Personal benefits | **“Why do you vape?” (Multiple-response options)**  23/41 reported “for pleasure” |  | Enjoyment   - Comments pertaining to enjoying vaping due to qualities such as flavour, and responses stating how the flavours of vaping were the only thing preventing them from using cigarettes again. | *Expansion*   - Participants describe feelings of enjoyment and comfort when vaping, which corresponds with reports of vaping “for pleasure” endorsed by 56% of participants. - This evidence suggests that vaping is eliciting pleasure, and may be explanatory of vaping behaviour (type of substance, flavour, frequency of vaping). |
| Personal benefits | **QVC item: “I will vape as soon as possible” (on a scale of 1 to 7, which denote strongly disagree and strongly agree, respectively, and 4 denotes “neither agree nor disagree).**  Range: 1-7  Average response: 3.76 | n=13  7 female, 6 male  **Substances vaped:**  Cannabis only, n=5  Nicotine only, n=5  Nicotine & Cannabis, n=3 | Independence   - Comments related to regaining independence over the time spent in acquiring nicotine and managing cravings.   Control   - Comments related greater control over their consumption | *Congruence*   - Participants describe how vaping allows them to have greater control over their consumption of nicotine and independence in choosing when to vape. This corresponds with average neutral responses to needing to vape as soon as possible. - Taken together, this evidence suggests that vaping may be associated with cravings that do not limit a user’s independence. |
| Personal benefits | **QVC item: “Nothing would be better than vaping right now” (on a scale of 1 to 7, which denote strongly disagree and strongly agree, respectively, and 4 denotes “neither agree nor disagree).**  Range: 1-7  Average response: 3 |  | Independence   - Comments related to regaining independence over the time spent in acquiring nicotine and managing cravings. | *Congruence*   - Participants describe vaping permitting greater independence over their time and ability to engage with other activities, which corresponds with general disagreement that vaping would be better than partaking in other activities. |
| Vaping and Smoking Reduction | **Number of cigarettes per day**  Mean 10.11 per day (SD 6.11) | N=20  13 female, 7 male  **Substances vaped:**  Cannabis only, n=2  Nicotine only, n=10  Nicotine & Cannabis, n=7  Water-flavour only, n=1 | Reduce smoking   - Comments specifically describing the motivation to vape in order to reduce the number of combustible cigarettes consumed. | *Discordance*   - Participants describe vaping supporting them in reducing the number of cigarettes they consume per day. - Estimates of daily smoking within OUD patients who do not vape suggest rates of 14-15 cigarettes per day (POST). - The number of cigarettes smoked per day by vapers does not differ greatly from those who do not vape, contrasting with participant beliefs and suggesting that vaping is supporting minimal reduction. |
| Vaping and Smoking Reduction | **Number of cigarettes per day among those reporting using e-cigarettes to reduce cigarette use vs. those who did not report this motivation for use.**  Mean 10.4 cigarettes per day vs. 11 cigarettes/day |  | Control smoking cravings   - Comments describing the motivation to vape to help avoid combustible cigarette cravings.   Reduce smoking   - Comments specifically describing the motivation to vape in order to reduce the number of combustible cigarettes consumed. | *Discordance*   - Participants perceived vaping to support them in reducing the number of cigarettes smoked per day. - This diverges from quantitative data shows near equal daily cigarette consumption between those who reported using vaping to reduce cigarette use these perspectives and those who did not. - This difference signals that despite perceptions that cigarette consumption is being reduced, vaping is not, on average, resulting in reduction. |
| Vaping and smoking cessation | **Number of participants who do not use cigarettes vs. use cigarettes (within the group of individuals reporting vaping as a smoking cessation tool)**  11 reporting no current smoking, 23 current smokers | n=34  20 female, 13 male, 1 nonbinary  **Substances vaped:**  Cannabis only, n=5  Nicotine only, n=20  Nicotine & Cannabis, n=8  Water-flavour only, n=1 | Smoking cessation   - Comments specifically discussing the need or want to stop smoking using vapes/e-cigarettes. | *Discordance*   - Participants perceived vaping to be helpful in quitting cigarette smoking, which contrasts with data showing that among those which reported this perspective (n=34), 68% continue with daily cigarette smoking. - This divergence suggests that vaping is ineffective in smoking cessation, and is contributing instead to dual use of nicotine. |
| Vaping and smoking cessation | **Mean number of years vaping**  Mean 4.58 years vaping |  | Smoking cessation   - Comments specifically discussing the need or want to stop smoking using vapes/e-cigarettes.   Vaping is a superior smoking cessation tool   - Comments describing vaping as a way to reduce or stop smoking, and the belief that vaping is more successful than alternative smoking cessation tools on the market. | *Discordance*   - Participants reported vaping to be a smoking cessation tool, suggesting it to be superior to alternative cessation tools. - Beliefs of superior cessation are unsupported by evidence which shows that on average, participants have been vaping for 4.5 years, and many remain smoking. - This disagreement provides poor evidence for vaping as a smoking cessation tool. |
| Vaping and substance use | **“Why do you vape?” (multiple response option)**  Response:   - 20 reported using vaping as a means of coping with substance cravings of various substances - 12 for tobacco cravings - 2 cannabis cravings - 3 crack/powder cocaine - 5 opioids (heroin, fentanyl) - 2 methadone | n=21  11 female, 8 male, 1 non-binary  Cannabis only, n=2  Nicotine only, n=12  Nicotine & Cannabis, n=5  Water-flavour only, n=1 | Minimizes cravings/engagement with substances of abuse drug cravings   - Vaping assists with or is used for the reduction in the use or in the cravings and habits associated with drugs other than methadone (i.e. crack, cocaine etc.). Vaping acts as a coping mechanism to support abstinence. | *Congruence*   - Vaping was perceived to be helpful in managing cravings for illicit drugs. - Participants perceptions corresponded with 20 of 41 participants reporting “managing cravings for substances” as a reason for why they vape, mentioning not only cravings for tobacco, but also substances like cannabis, crack or powder cocaine, and opioids like fentanyl and heroin. |
| Vaping and substance use | **“Why do you vape?” (multiple response option)**  Response:  20 reported using vaping to avoid/substitute for other substances. |  | Minimizes cravings/engagement with substances of abuse drug cravings   - Vaping assists with or is used for the reduction in the use or in the cravings and habits associated with drugs other than methadone (i.e. crack, cocaine etc.). Vaping acts as a coping mechanism to support abstinence.   Habit replacement   - Comments describing how vaping helps with oral fixation and sating hand-to-mouth impulses typical to cessation. | *Convergence*   - Participants found vaping to be helpful for managing cravings for illicit substances, which parallels quantitative reports of using vaping to avoid/substitute for other substances. - This suggests that vaping is perceived by participants to impact their other substance cravings |
| Vaping and substance use | **Urinary toxicology screens (percent positivity)**  Results:   - 18.75% positivity for opioids - 6.25% positivity for benzodiazepines - 37.50% positivity for amphetamine - 28.57% positivity for cocaine - 33.33% positivity for methamphetamine |  | Minimizes cravings/engagement with substances of abuse drug cravings   - Vaping assists with or is used for the reduction in the use or in the cravings and habits associated with drugs other than methadone (i.e. crack, cocaine etc.). Vaping acts as a coping mechanism to support abstinence.   Habit replacement   - Comments describing how vaping helps with oral fixation and sating hand-to-mouth impulses typical to cessation. | *Discordance*   - Participants mentioned vaping helped with habit replacement and physical cravings related to illicit drugs. - Participant views contrasted with objective urine screen data which showed positive screens for not only the specifically mentioned substances, but also for other illicit substances. - This disagreement generates concerns that vaping is not an effective method for curbing cravings or coping, and may instead be resulting in another addiction. |
| Vaping is socially motivated | **In which of the following situations do you most typically vape?**  6 (14.6%) participants reported vaping most typically around others (two or more others) | n=15  10 female, 4 male, 1 nonbinary  **Substances vaped:**  Cannabis only, n=1  Nicotine only, n=9  Nicotine & Cannabis, n=5 | Social value   - Comments related to improved social interactions or improved perceptions of self from members of one’s social group after commencing vaping. Comments surrounding initiating or continuing vaping due to vaping occurring within their social group. | *Congruence*   - Participants reported social situations motivating them to start and continue vaping, corresponding with 14.6% of participants reporting that they most typically vape around others. - Almost 1 in 7 participants vape most typically around others, meaning that others may vape around others, but this is less typical. This supports a strong social component for vaping, impacting vaping behaviour. |
| Vaping is socially motivated | **Why do you vape?**  8 (19.5%) reported vaping because others around me are also vaping. |  | Social value   - Comments related to improved social interactions or improved perceptions of self from members of one’s social group after commencing vaping. Comments surrounding initiating or continuing vaping due to vaping occurring within their social group. | *Congruence*   - Participant views on deriving social benefit from vaping, consistent with participants also reporting they vape because others around them are vaping. - Both qualitative and quantitative data suggest that vaping can have a social component, and that social settings or interactions can motivate vaping. |
| Vaping and health | **QVC question: “I am missing vaping right now”**  3.7 mean response | n=33  17 female, 15 male, 1 nonbinary  **Substances vaped:**  Cannabis only, n=7  Nicotine only, n=16  Nicotine & Cannabis, n=9  Water flavour only, n=1 | Vaping is addictive   - Comments discussing vaping as addictive or potentially addictive, and/or descriptions of the addictive attributes of vaping when explaining their experiences.   Vaping is not addictive, and I use it to stop smoking.   - Comments describing using vaping as a means to stop smoking, while not considering vaping itself to be addictive. | *Congruence*   - Some participants described vaping as potentially addictive and harmful, while others shared perspectives that vaping was nonaddictive, and something they could discontinue easily. - Participants’ divided views align with participants level of agreement on “missing vaping” to disagree with neutral feelings toward the idea of “missing vaping.” |
| Vaping and health | **QVC Score**  Mean (SD)= 36.82 (16.13) |  | Vaping is addictive   - Comments discussing vaping as addictive or potentially addictive, and/or descriptions of the addictive attributes of vaping when explaining their experiences. | *Congruence*   - Participants described vaping as potentially addictive and thereby harmful toward health, which appears to agree with moderate levels of cravings within this population (max score: 70, min score: 10) - Moderate cravings scores are congruent with views that vaping may be addictive. |
| Vaping and health | **Comorbid Conditions**  51.2% report at least one mental health (anxiety, mood disorders, stress disorders) |  | Vaping has positive effects on health   - Comments describing that the impacts of vaping on health, mental health in particular (i.e. anxiety, stress etc.) | *Discordance*   - Well over half of the participants in this sample reported some form of positive health effect of vaping, particularly valuing the effect of vaping on mental health through easing stress and anxiety. - Participant perspectives appear to contrast with data on co-morbidities, which show that over half endorse some type of mental health diagnosis, though this data is does not reflect possible improvements in symptoms. |
| Vaping and health | **“How often do you vape?”**   - Everyday vaping (n=35, 85.4%) - Every other day (n=2, 4.9%) - 2-3 times per week (n=3, 7.3%)   1-2 per month (n=1, 2.4%) | n=23  15 female, 8 male  **Substances vaped:**  Cannabis only, n=6  Nicotine only, n=12  Nicotine & Cannabis, n=5 | Vaping is addictive   - Comments discussing vaping as addictive or potentially addictive, and/or descriptions of the addictive attributes of vaping when explaining their experiences.   Vaping is not addictive, and I use it to stop smoking.   - Comments describing using vaping as a means to stop smoking, while not considering vaping itself to be addictive. | *Congruence*   - Some participants described vaping as potentially addictive and harmful, while others shared perspectives that vaping was nonaddictive, and something they could discontinue easily. - Data on frequency of vaping shows that approximately 85.4% of participants vape every day, and most of the remaining vape at least multiple times per week. - Frequency data is congruent with perspectives that vaping is addictive and does not provide support for perspectives that vaping is not addictive. |
| Vaping and MOUD | **Urine toxicology screens, percent positivity for opioids at time of interview**  16.22% positivity for opioids | n=10  5 female, 4 male, 1 nonbinary  **Substances vaped:**  Cannabis only, n=4 Nicotine only, n=2  Nicotine & cannabis, n=4 | Vaping does not impact MOUD  Comments describing neutral/no effect of vaping on MOUD treatment.  Vaping has some effects on MOUD   - Comments describing positive effects on MOUD, and indirect benefits of vaping on MOUD treatment success. | *Congruence*   - Participants either no effect of vaping on MOUD or mild effects, mentioning it may help with tapering dose or coping with withdrawal symptoms, and thereby supporting treatment success. - The modest perceived effects are congruent with urine screens which are positive for opioids, suggesting that any perceived effects may be inconsistent and unreliable. |
| Vaping and Youth | **Mean age of the sample**  Mean (SD) = 40.26 (12.23) | n=7  3 female, 4 male  **Substances vaped:**  Nicotine only, n=5  Nicotine & cannabis, n=1 Water flavour only, n=1 | Vaping is popular among young people   - Discussion of vape as something that is most common within a younger age group. | *Discordance*   - Participants often discussed perceptions of vaping being common among youth, specifically students and young adults, which is incongruent with the mean age of the sample. - This inconsistency may be explained by perceptions of vaping in young people in the general population, and may not be reflective age-related trends within the OUD population. |
| Vaping and Youth | **Mean age when first tried vaping**  Mean (SD) = 33.95 (12.70) |  | Vaping is popular among young people   - Discussion of vape as something that is most common within a younger age group. | *Discordance*   - Perceptions of vaping being popular among youth disagrees with the mean age of first trying vaping within this sample, which suggests that, on average, first attempts at vaping occur during the early to mid-thirties. - This inconsistency may be explained by perceptions of vaping in young people in the general population, and may not be reflective age of first trying vaping within the OUD population. |
| Vaping and Youth | **Mean age when first started vaping regularly**  Mean (SD)= 34.85 (12.38) |  | Vaping is popular among young people   - Discussion of vape as something that is most common within a younger age group. | *Discordance*   - Perceptions of vaping being popular among youth conflicts with the mean age of first initiating regular vaping, which suggests regular vaping begins around age 35. - This inconsistency may be explained by perceptions of vaping in young people in the general population, and may not be reflective age of regular vaping within the OUD population. |
| Vaping and Youth | **Flavour**  Of the 8 participants younger than 30 years, 25% reported using flavours. Of the 33 participants, 36% of participants reported vaping flavoured products. |  | Vaping flavours are attracting youth   - Discussion of how vaping flavour options are attractive to young people, including flavour options without nicotine or cannabis components. | *Discordance*   - Participant perceptions suggest that flavours involved with vaping are attracting youth. - Data shows a higher proportion of those older than 30 are using vape flavours than though below 30. - If participant perceptions were accurate, proportions of those vaping flavors would be higher in the younger sub-group. This divergence suggests that perhaps these beliefs and observations may apply to the general population, and are less explanatory of flavour trends within the OUD population. |
| Vaping to get high | **Cannabis vaping in the sample**  33% vaping cannabis products in the full sample | n=7  3 female, 3 male, 1 nonbinary  **Substances vaped:**  Cannabis only, n=3  Nicotine & cannabis, n=4 | Vaping as a way to get “high”   - Comments related to vaping in order to achieve a “high” or feeling of euphoria, typically discussed in the context of cannabis vaping. | *Congruence*   - Motivations for vaping to get high are congruent with data showing that nearly 33% of the sample vapes cannabis products. - This suggests that participants are motivated to vape in order to get high, and are consuming cannabis. |
| Vaping to get high | **“Why do you vape?” (Multiple response options)**  13 participants reported “to get a high” as a reason for vaping |  | Vaping as a way to get “high”   - Comments related to vaping in order to achieve a “high” or feeling of euphoria, typically discussed in the context of cannabis vaping. | *Congruence*   - Motivations for vaping to get high are in agreement with participants including “to get high” as a reason for vaping in 32% of participants. |
| Vaping to get high | **Urinary toxicology screens (percent positivity)**  33% positivity for cannabis |  | Vaping to get “high”   - Comments related to vaping in order to achieve a “high” or feeling of euphoria, typically discussed in the context of cannabis vaping. | *Congruence*   - Motivations for vaping to get high are congruent with data showing 33% positivity for cannabis within the sample. |
