# Supplementary material for: Effects of vaping on physical and mental health in at-risk populations (VAPE): mixed-methods study of motivations for and perspectives on vaping in patients with opioid use disorder
Source: BJPsych Open. 2025 Apr 2;11(3):e75. doi: 10.1192/bjo.2025.6 (PMC12052573; doi:10.1192/bjo.2025.6)
Supplement: D’Elia et al. supplementary material 6 — D’Elia et al. supplementary material [file S2056472425000067sup006.docx]

**Appendix F: Codebook for Themes on Motivations and Perspectives of Vaping in the OUD population**

| Themes | Node | Description | Examples |
| --- | --- | --- | --- |
| 1. Perceived convenience | Convenience/ease of use | Comments related to the ease of use of vapes/e-cigarettes in comparison to cigarettes and other forms of nicotine intake. | - “I don’t mind using the vape inside, so that’s nice.” (Case 13, M/36) - “Um.. cause… cause like I buy a cart- like I’ll buy a cartridge and then I’ll buy a pack of cigarettes, and if I don’t have the money, then I just use the vape.” (Case 1, F/36) - “Um, the convenience of it, and that it doesn’t smell like cigarettes and stink up the house?” (Case 3, F/33) - “And sometimes it’s just having a couple of inhales of the vape- the vapourizer is quicker than trying to smoke a half cigarette.” (Case 5, M/25) - “Yeah, I’m not having to go outside every time to, to have a cigarette. I don’t mind using the vape indoors.” (Case 13, M/36) - ““Um, I don’t smoke in the house, where I live, so I don’t always wanna go outside for a cigarette, and I keep a e-cigarette around just so, you know, just to be handy. Um, to, to vape when I can’t get to a cigarette outside.” (Case 3, F/33) - “That’s $15.75 a day, plus I was smoking a pack and a half, so. It, it was getting too much. I couldn’t afford it.” (Case 6, F/29) - “I like it because it’s um you don’t have to do anything you know what I mean? Like all I have to do is open the the pen up and start puffing on it. I don’t have get it all the.. you know to get papers ready and get crap all over my clothes and you know what I mean? Like it.. no.. there’s no mess it’s just you just open the package up and you start puffing.” (Case 31, F/52) |
|  | Cost | Comments discussing or initiating because it is cheaper or because others have recommended it as a method for cost savings. | - “Um, yeah so like smoking cigarettes was getting so expensive, uh, when I started smoking I remember like, a pack of smokes was like, well you getting a thing called presto packs, they were like $3 and you have to put the filters on yourself. Um, and so the last time I bought a pack of smokes it was like $20, so it was just like, I mean, some people go to the reservation, and you can get them for a lot cheaper there, but uh-uh I just couldn’t afford it.” (Case 27, F/39) - “Uh, just, she was like, trying to quit smoking, and she was like, hey, “you should try this”. It- it made her feel healthier, it was cheaper, and so, she kind of, she actually gave me my first vape. Uh, my first rig, and was just kinda like, “oh, here, try this and hopefully you know, it’ll help you quit smoking, save you some money” (Case 27; F/39). - “Yeah, I think it uh it has positive for sure like uh, my lungs are starting to clear up. I’m not coughing every morning and coughing up a lot of phlegm. Um, it’s uh, it’s slowing down the smoking, less money – doesn’t cost as much. Lasts twice, three times, four times as longer than cigarettes do. I don’t have to travel all the time to get them. I just gotta buy a bottle of juice and it lasts quite a long time. (Case 19; M/64) - “Because, cigarettes were though, the price on cigarettes – weren’t getting them from the reserve, the price was getting out of control.” (Case 19; M/64) |
| 2. Perceived pleasure | Comfort | Comments detailing pleasant experiences with vaping related to social benefits (i.e., smell) and self-care habits such as relaxation, particularly when compared to cigarette smoking. | - “Just, I kinda got used to it, a little bit? Um, with the little setting when I was with my ex-boyfriend, so, I just kinda picked up the habit with him and I just continued to, you know, buy one once in a while. To have with me” (Case 3, F/33) - “Yeah. Like, my hands didn’t smell afterwards. And stuff…” (Case 1, F/36) - “Um, it looked like a cleaner alternative to cigarettes. Made you smell good.” (Case 11, M/43) - “Um, yeah, I have like, uh, uh, I only vape like maybe once a day if it’s like nighttime and I’m watching a movie … Um, to, to vape when I can’t get to a cigarette outside. Or when I don’t feel like getting up.” (Case 3, F/33) - “Sometimes it helps with relaxation when I’m at home and I don’t wanna move, and I can just relax and I have my e-cigarette with me and uh, veg on the couch.” (Case 3, F/33) - “So it was like, I hadn’t completely shifted my mindset yet to replace like one thing with another, and then by the time – what happened was I went somewhere for the weekend and there was no cigarettes, and all I had was the vape, and by the time I got back to where my cigarettes were, and I lit one up, and it tasted horrible.” (Case 27, F/39) - “So, now I just sit on my couch and kinda hang out. Which, is almost worse, cause I do like have this thing in my hand all the time.” (Case 28, M/30) - “Uh the smell, uh I say that I work in restaurants right, so it’s not like you go outside smoke come back in and not stink so, for certain reasons yes.” (Case 17, M/41) |
|  | Enjoyment | Comments pertaining to enjoying vaping due to qualities such as flavour, and responses stating how the flavours of vaping were the only thing preventing them from using cigarettes again. | - “Yup, um, so I had smoked for probably like, 25 years, when I started, or when I first got it, and had no intentions on quitting. But um, but I ended up quitting. I just, I really liked -- the flavour is much better, I enjoyed the “pull” over a cigarette “pull”. Um, the house wasn’t as disgusting, as it was with two of us smoking in there.” (Case 18, F/41) - “Any fruit flavours I tried. You know, grape, lemon, umm a lot of the citrus flavours I enjoyed, like apple, um you know, you name it, plum, you know, plums, anything, anything that was fruit flavoured. It was all I bought, exclusively, 100%.” (Case 19, M/64) - “Like, um, so, one of my buddies, was like oh you know, there’s like, there’s good flavours. And like, kind of slowly convinced me. Um, and yeah, like. The second that I picked this thing up, I-I stopped smoking cigarettes, that way.” (Case 28; M/30) |
| 3. Perceived Agency | Control | Comments related greater control over their consumption | - “Yeah. So, like, I mean, I just like the ability to, um, begin, and and stop whenever I choose.” (Case 4, F/41) - “Um, and, if like, yeah I fi-, cause now I’m not doing it all, you know what I mean? When you light a cigarette, you smoke a full cigarette.” (Case 4, F/41) |
|  | Autonomy | Comments related to regaining independence over the time spent in acquiring nicotine and managing cravings. | - “Um, I really, don’t do much research into that kinda stuff, like I just kinda go with my day and go with the flow, and um, for me, not to have to smoke a full cigarette or not to – like I just find that it, it fixes my craving, um, within one puff. Which, as I say, one puff is like 5 times a day, that’s like smoking [emphasis] a cigarette in a day.” (Case 4, F/41) - “Oh yeah, I have [tried to quit]. It was very easy, I just put it down and I don’t touch it.” (Case 4, F/41) - “And sometimes it’s just having a couple of inhales of the vape- the vapourizer is quicker than trying to smoke a half cigarette.” (Case 5, M/25) |
| 4. Perceived smoking reduction | Control smoking cravings | Comments describing the motivation to vape to help avoid combustible cigarette cravings. | - “Yeah, I ran out of cigarettes and he was like “here try this”, so I tried it and it, um, helped with um, the cravings, and then – I still smoked, but you know I would try once in a while. Or do it with him.” (Case 3, F/33) - “Like I don’t see, it’s – but then again I’d have to smoke cigarettes so.” (Case 9, M/31) |
|  | Reduce smoking | Comments specifically describing the motivation to vape in order to reduce the number of combustible cigarettes consumed. | - “I’m more addicted to cigarettes, like I’ve smoked cigarettes since I was 14 years old. And with vaping, I usually just uh, I usually just do it to try and see if I can go a day or – a couple days without smoking cigarettes.” (Case 5, M/25) - “Uhhh, well it all depends right, it’s been off and on for the last 10 years or so. Since I started vaping. So, there’s been times where I’ll smoke more than I vape, right?” (Case 7, M/33) - “Somewhat, I cut down, but not. Like.” (Case 10, F/33) - “Yeah, like, not completely I guess, like I still smoke cigarettes and I still vape. But…” (Case 5, M/25) - “It was usually a matter of going back to cigarettes, or going down on the cigarettes and choosing the vape more.” (Case 11, M/43) |
| 5. Vaping is perceived to be a smoking cessation tool | Smoking cessation | Comments specifically discussing the need or want to stop smoking using vapes/e-cigarettes. | - “So, um, like I’d say the first month I was still smoking, like cigarettes, strongly. But after that, and I, um, I almost went three months straight without, no cigarettes.” (Case 7, M/33) - “And, um, I wanted something to, you know what I mean? You, you always have those things where you put a cigarette in your mittens, your fingers, and not light it. Or people have their different ways.” (Case 4, F/41) - “It, it, it… it just, it helped me a lot with quitting smoking, and if it helped me, it could help other people. You know, I had a really hard time with quitting drugs, um, and so I thought, quitting smoking would never [emphasis] be an option.” (Case 6, F/29) - “Sure. Uh, uh I vape, um, I vape as a tool to- to- to- quit cigarettes, to quit cigarettes. So, basically I vape to- to- to- um, yeah, to help the void from cigarettes.” (Case 9, M/31) - “I just, I don’t know, I use it as a replacement for cigarettes, and um I guess I’m addicted to it now.” (Case 16, M/45) - “It was a substitute for cigarettes.” (Case 17, M/41) |
|  | Vaping is a superior cessation tool | Comments describing vaping as a way to reduce or stop smoking, and the belief that vaping is more successful than alternative smoking cessation tools on the market. | - “Yeah. All the nicotine patches, the little puffer things, yeah, […] nothing worked [as a cessation tool].” (Case 12, F/46) - “Um, yeah because I had heard of it as an alternative to cigarettes. It was all because I wanted to quit cigarettes so I was looking for options, and there hasn’t ever been many options other than like, you know, nicotine gum and stuff, and that stuff just doesn’t work.” (Case 9, M/31) - “Um. Tell you a little bit about my experience. So, um I guess my two cents on that is um, uh, I I I pri-, I don’t know I have a strong belief that it’s a great tool for quitting cigarettes, so my experience is is is overall decent. Like, um […] Um, it’s good, it’s really good. Uh, I don’t think, in comparison to the amount of cigarettes I smoke, the amount I vape is nil in comparison to what I smoke for cigarettes.” (Case 9, M/31) |
|  | Vaping is not an addiction | Comments describing that vaping is not seen as something that is addictive or something they wish to stop. | - “But uh, again there’s that whole hand to mouth thing also, so that’s why I’m hesitant to say I’ll withdraw it [vaping] altogether.” (Case 13, M/36) - “No, I would never, I wouldn’t even see vaping as something that I’d need to quit. Like wouldn’t be a problem.” (Case 11, M/43) - “Because… no, no. I haven’t tried to quit vaping. Uh, its an interesting question because um, I never thought of it that way. I feel like vaping is almost one of those things where I, if I didn’t wanna vape I just wouldn’t.” (Case 9, M/31) - “Um, because I’ve never really been a regular, regular vaper I guess? So I never saw it as something I should try and quit. (Case 3, F/33) |
|  | Vaping is different than cigarettes | Comments stating that vaping is different, better and healthier than cigarettes, offering a multitude of benefits compared to combustible cigarette smoking./smoking cannabis | - “Um, it looked like a cleaner alternative to cigarettes. Made you smell good.” (Case 11, M/43) - “Uh, just, for the health, and the not having to inhale the smoke, and uh, for the smell.” (Case 13, M/36) - “There’s a little bit of a different effect. Like not a different effect, but there’s a little – it just, I can’t really explain it, it just. It um, it tastes a little bit different?” (Case 4, F/41) - “That, and it’s, it’s a good, it’s a good delivery system for the products I want to consume.” (Case 8, M/36) - “Um, I wouldn’t even say a lowered intake, just a different method of intake, uh, but no that’s definitely the number one reason.” (Case 13, M/36) - “You know? Like I mean, I just find, for myself, um, its, to me its healthier, to me it doesn’t smell, you know what I mean? There’s a lot of pros to vaping for me, um, especially when I have children.” (Case 4, F/41) |
|  | Nicotine Satiation | Comments expressing desire to vape to satisfy cravings for nicotine. | - “Um I didn’t think about that [health effects of vaping], anything about my health at all, it just helped me stop smoking…” (Case 12, F/46) - “But you’re still getting your fix of the nicotine too, so it actually just kinda kills two birds with one stone” (Case 4, F/41) - “Yeah, I ran out of cigarettes and he was like “here try this”, so I tried it and it, um, helped with um, the cravings, and then – I still smoked, but you know I would try once in a while. Or do it with him.” (Case 3, F/33) - “Why I do it. To keep my addiction at bay. Cause I’m – I can get pretty grouchy if I’m – as anybody – without – I go into my nic-fit.” (Case 28, M/30) - “Yes. It’s just it’s just only if I’m out of cigarettes I’ll pick it up.” (Case 30, F/40) - “Well, I didn’t try to quit, it just happened that I quit and smoked more cigarettes than vaping.” (Case 15, F/59) |
|  | Vaping is a maintenance treatment | Comments likening vaping to maintenance treatment for discontinuation of cigarettes, specifically methadone treatment. | - "But I mean I vape nicotine for pretty much to continue like it’s like how methadone like keeps you from relapsing on opiates. It’s the same thing, like vaping keeps me from relapsing on cigarettes. Cause if I have no nicotine, I’m more likely to be anxious…” (Case 6, F/29) - “Yeah, it’s the same concept, basically, its like, my uh – the vaping is like the methadone for my smoking, um – that’s what it reminds – although I’m having a lot harder time tapering off methadone, than I am off the nicotine. So.” (Case 27, F/39) - “Um, not really. Just that it it has it it’s replaced smoking in a lot of situations. Um, so I I I see it as a positive thing. Um as a tool. As a tool to get off of the the smoking kind of like what methadone would be for opiates. I, um, I’m using it in the same manner. So I’ve enjoyed my experience vaping, um, and uh, it is it is definitely in my mind something temporary.” (Case 41, F/39) - “But I mean I vape nicotine for pretty much to continue like it’s like how methadone like keeps you from relapsing on opiates. It’s the same thing, like vaping keeps me from relapsing on cigarettes. Cause if I have no nicotine, I’m more likely to be anxious…” (Case 36, 24/N) |
|  | Vaping is addictive and I use it to stop smoking. | Comments describing using vaping as a means to stop smoking, while acknowledging vaping to be addictive. | - “I just, I don’t know, I use it as a replacement for cigarettes, and um I guess I’m addicted to it now.” (Case 16, M/45) - “Um, overall, I’ve had uh – I’ve had a good experience with it. I’ve like um, I just was saying this earlier, it uh, I find its made me feel a lot better, in my health, and just, um, yeah, its its, it’s been really handy to come off of smoking. Uh, to have it as a backup. I, I do think though it, its something that I would like to quit doing. Like I don’t wanna do this the rest of my life. So.” (Case 27, F/39) - “Um, but I guess, I would just say like, I think that there is a positive side to it. Um, because I think it is less - I think if you’re doing it in a way to help you quit smoking, and you’re not just like, trying to get big clouds, or not really nicotine levels, and you’re – I think it has been really helpful for a lot of people” (Case 27, F/39) - Like, I’m already addicted to nicotine, but it’s not like I’m, you know, not smoking nicotine? […] When I’m vaping. I’m still doing that, so. I’m aware of that. (Case 24; F/33) |
|  | Vaping is not addictive, and I use it to stop smoking | Comments describing using vaping as a means to stop smoking, while not considering vaping itself to be addictive. | - “Um, I don’t find it, I don’t need it – I, I only use it when I, when I feel like using it. I don’t have like, uh, uh uh, a strong – it doesn’t have a strong hold on me, like I don’t need it every day.” (Case 29, F/53) - [Interviewer: “Is vaping something that, um, you view as problematic or something that you would ever want to try to quit?”] Participant: “Neh.” (Case 1, F/36) |
| 6. Perceived changes in substance use | Minimizes cravings/engagement with substances of abuse drug cravings | Vaping assists with or is used for the reduction in the use or in the cravings and habits associated with drugs other than methadone (i.e. crack, cocaine etc.). Vaping acts as a coping mechanism to support abstinence. | - “Well, to be honest with you, I was in active addiction, um, and um, uh, I went in and quit. I got clean. And when I went and got clean, I decided to quit smoking. Smoking weed, and quit- quit doing dope…And, um, I wanted something to, you know what I mean? You, you always have those things where you put a cigarette in your mittens, your fingers, and not light it. Or people have their different ways.” (Case 4, F/41) - “For sure. Absolutely. I have a lot of dreams about [crack cocaine] – and it’s weird because that was not my drug of choice, and, it’s weird that I get cravings where I’m wanting to smoke it, but when I do, I will smoke my vape, and honestly, right away, it’s just gone. And because, in my head I don’t want to ever go back to that lifestyle.” (Case 6, F/29) - “Um, yeah it like just keeps me from, like, being bored, stuff like… Takes my mind off of things.” (Case 1, F/36) - “Uh, the one reason is because I had been trying to get off of the fentanyl. And, if I couldn’t get it, it would take away the physical ummm, withdrawal symptoms that I was - uh – going through. Like the stomach aches, the bone aches. The, the, the overall creepy feeling that you get. The vaping would take that away.” (Case 29, F/53) - “Not at all, it’s not a social thing at all right now, it’s more so, yeah like boredom stress release, especially since I quit smoking other things which were kinda my coping mechanism.” (Case 14, F/18) |
|  | Habit replacement | Comments describing how vaping helps with oral fixation and sating hand-to-mouth impulses typical to cessation. | - “I think the big thing is that hand umm.. or the hand motion. That’s like the biggest thing for anyone who’s a smoker so whether that be like [inaudible] or tic tacs or anything.” (Case 35, F/20) - “Like for relaxation. And like I find that like having something in my hand is really helpful. Um. I mean there’s fidget toys for autistic people like myself but there is you know when you’re an adult like you really like “why are you playing with a toy”? You know what I mean.” (Case 36, N/24) - “Um, I wouldn’t even say a lowered intake, just a different method of intake, uh, but no that’s definitely the number one reason, but uh, again there’s that whole hand to mouth thing also, so that’s why I’m hesitant to say I’ll withdraw it altogether.” (Case 13, M/36) - “Um because I have an oral fixation.” (Case 15, F/59) |
| 7. Perceived lack of information about vaping | Knowledge acquisition | Comments related to the dearth of information on vaping and its effects (largely physical health effects) and the desire to learn more about the effects of vaping from external sources (media, doctors etc.)  . | - “Um, I am weary of vaping because there’s not that much – I’m not sure about the statistics on it? I know cigarettes cause cancer and stuff like that, but, I feel like vaping has gotta be – there’s gotta be some, some sort of medical downfall to vaping. Um, it’s just another toxin that we’re putting into our bodies. So, I figure there’s gotta be something bad about it, we just haven’t found out yet.” (Case 3, F/33) - “Hmm, not really, um, just that it’s not as good for you as people think it is” (Case 2, F/46) - [Interviewer: Do you think you would have started, um, vaping, even socially, uh, if you had known, earlier, about the effects on the lung?] Participant: “No ” (Case 2, F/46) - [Interviewer: Okay. And who told you, um, just out of curiousity, who, uh, mentioned – or where did you hear that it was bad?] Participant: “On youtube”. (Case 2, F/46) - “Um, I just, a question mark on negative health effects [unintelligible], but uh, I haven’t done too much research into it myself so.” (Case 13, M/36)   “It’d be interesting actually, what your research finds when it comes to that, like how many people it actually helped feel better, and how many people felt worse from it – “ (Case 27, F/39)  “It’s been around for, I think, 8 years or 10 years or something, but it’s still new in-in-in, as far as like what we know what it does to us.” (Case 27, F/39)  “…So that’s why I don’t do it as regularly like I initially wanted to to use it to quit smoking cigarettes but I didn’t um continue really because there’s not enough research about it.” (Case 30, F/40) |
| 8. Perceived social benefits motivate vaping behaviours | Stigma | Comments attributing perspectives of external sources, specifically related to the negative consequences of vaping, to stigma. Association of external information with stigma and misinformation, urging that the positive effects of vaping as a smoking cessation tool are yet to be discussed. | - “…I was really proud of myself for like so, and a lot of people around me were proud cause I had smoked cigarettes for 15 years. And, and then this crazy stigma came in with like how bad vaping was and then I started hearing ridiculous people talking about like – “Oh vaping is terrible, it’s even worse than cigarettes.” And then that really bummed me out, because uh I found like, it was such a good tool to help people save their lives from cigarettes. And it was all because uh, this whole mix up with people dying from it. But there was a stigma around – the research – cause I was into it, I did a lot of research, and it, it turns out that it had a lot to do with uh, vaping um THC and some type of knock-off cartridges from – that’s what was killing people. People, don’t understand that.” (Case 9, M/31) - “So, so, in combination with um, with the lockdown and everything, there was that, plus at that exact time uh, there was this huge stigma going on, there was, it was all over the news about like vaping being, like killing people. It was all over the news and media. And uh, a lot of stuff was happening and there was a lot of misinformation at the time.” (Case 9, M/31) - “That I’ve talked to. Um, so I – I don’t know how you can combine that into your research, as far – I just, would like people to be aware that it’s not – it’s not as, as bad as it’s kind been stigmatized as.” (Case 27, F/39) |
|  | Social value | Comments related to improved social interactions or improved perceptions of self from members of one’s social group after commencing vaping. Comments surrounding initiating or continuing vaping due to vaping occurring within their social group. | - “Yeah. Yeah, um, totally, and that was like, that was huge for me. Like even people that knew me were like holy like – um, they just even would remark like “oh,” I think it was my neighbour that said, I would sit outside with like a cigarette and my coffee in the morning. And my neighbour was like: “Oh I don’t hear you coughing anymore” and it just made me realize that even other people were aware of like – you know, me sitting out there in the morning, coughing, like – I didn’t – I wasn’t even aware of it. (Case 27, F/39) - “Um, he doesn’t like me smoking so he suggested I started to vape.” (Case 14, F/18) - “Well I vape to kind of slow down my tobacco use, um, and I-I like the way it doesn’t make my clothes smell, it doesn’t make my hair smell. Um, it looks a lot more friendlier around my kids.” (Case 24; F/33) - “Oh okay, uh, um, it’s – ju – really it’s been – it’s actually positive. Uh, one because, at this age, you’re sick and tired of the, of the smoke, like you-your just trying to just s–s- like um, discreetly, use. You know, like you’re not trying to tell the world, you know, banners all over the place – I’m not an activist, so I try to keep – I’m very low key.” (Case 42; M/65) - “It’s a little cleaner. Um, in, in society now, it seems to be more accepted.” (Case 37; F/52) - “Um, yeah well it felt like cooler more like technology, like futuristic technology kind of. Like I could tell that it was the future of nicotine use. Um in a lot of ways. And I I felt like uh when I was assembling it I felt like uh it definitely was clunky kind-of designed and I felt like it could have been improved upon. But like um overall I I thought like it was pretty cool. I felt like cool when I was vaping which sounds so stupid to say cause I mean you look back on your high school years and think none of that was cool, heh. But, I mean.” (Case 36, N/24) |
| 9. Perceived positive health effects of vaping | Vaping is healthier for me than cigarettes | Comments describing the impact of vaping on physical health (i.e. decrease negative lung effects, taste and smells is coming back, more energy etc.) in comparison to effects of cigarette smoking. Comments describing vaping as better for their health than cigarettes, as evidenced by improvements in physical health, and vaping as more congruent with a healthier overall lifestyle. | - “It [coughing and breathing] got hugely better, cause like I’m not smoking a half and a pack a day.” (Case 6, F/29). - “You know? So for me, I feel like I breathe better…I don’t cough as much.” (Case 4, F/41) - “Less phlegm, less phlegm in my throat, for coughing. Like, there’s, smoking causes me to cough more.” (Case 11, M/43) - “So, for me, it’s been a positive, its helped my health because I am not smoking a lot of cigarettes.” (Case 9, M/31) - “And then, and then I also, throughout like that whole period of time, started trying to like, be more healthy overall. So like, I started doing yoga, and stuff, and I totally noticed a – I noticed a huge difference in just like – um, cause breathing was such a big part of it. And I noticed my lung capacity changed, my um, just, yeah, so much about like my physical, I just – I know people are like oh it’s still not healthy, and I understand that, like, vaping’s still not considered healthy, but personally, its – its been like 80% more healthy for me.” (Case 27, F/39) - “Oh, I was just gonna say, I used to get pneumonia a lot when I smoked […] But I haven’t, since I started vaping.” (Case 27, F/39) - “Um, well I just find it’s, it’s, um less like I breath better when I’m vaping, um I like it better than the cigarettes, like the cigarettes seem to be really harsh and really hard on my throat where this doesn’t seem to be.” (Case 16, M/45) |
|  | Vaping has positive effects on health | Comments describing that the impacts of vaping on health, mental health in particular (i.e. anxiety, stress etc.) | - “So, it’s one of those things where, if I’m stressed out, or, if I’m driving, or, any other time that I would, you know, normally light up a cigarette, I would just, I would just vape. And I’d like maybe take one or two puffs and then I’d put it away and then its, you know – and then its done for…” (Case 4, F/41) - “It’s just something that calms the, you know what I mean? Calms the stress, calms the, yeah.” (Case 4, F/41) - “Uh, yeah. Sometimes it helps with relaxation when I’m at home and I don’t wanna move, and I can just relax and I have my e-cigarette with me and uh, veg on the couch” (Case 3, F/33) - “Oh yeah, there’s definitely positive effects, you have a lot more energy, I have a lot more energy. My taste and my smell is coming back, and everything so…” (Case 7, M/33) - “It was calming, it, uh, relaxed me, and uh, I just, I – it made me feel – it made me feel, happy.” (Case 29, F/53) - "Yeah, it’s had positive affects in like stressful situations or boredom, it helps me from doing other things when I’m bored and stuff like harder drugs, I guess.” (Case 14, F/18) |
|  | Vaping as a health risk mitigation strategy | Comments related to motivation to vape to help avoid the risks and harms associated with alternative methods of consumption. | - “So, when I realized that I could get my daily THCs for cheaper and easier and less - health – like- harm reduction wise.” (Case 8, M/36) - “Uh, just, for the health, and the not having to inhale the smoke, and uh, for the smell.” (Case 13, M/36) - “Umm, because it’s the safest way I have to consume the both, the cannabis and nicotine right now, --- and I’m all about harm reduction.” (Case 18, F/41). - “Umm, yeah it does for sure. Like it reduces my stress levels, um, so I don’t always get to that point where I’m gonna be craving something […] Kinda prevents, preventative measures” (Case 18, F/41). - "Ummm, yeah, it it does for sure. Like it reduces my stress levels, um, so I don’t always get to that point where I’m gonna be craving something. […] Kinda, prevents, preventative measures.” (Case 18, F/41) - “And say you had asthma conditions and stuff, and you still wanted to, to, s-smoke, you were definitely better to be vaping, uh, then then, smoking, if you had any kinda asthma condition. And you couldn’t get off that that urge to, you know that Oedipus urge to smoke. You know?” (Case 19, M/64) |
| 10. Perceived negative health effects of vaping | Negative physical health symptoms when starting vaping | Harsh or strong physical health experiences with vaping when initiating use as participants tried to find nicotine levels that were ideal for them | - “It was, it was, um, I don’t know, it was a bit too strong. Like I couldn’t, I wasn’t used to, it took me a while to find the right nicotine strength…. I kept trying til I found what worked for me.” (Case 11, M/43) - I found at first, it uh, if I was smoking it too much, I felt nauseous, and I would get headaches. Um, so I slowed down on it. Um, and still if, if I take too many puffs of it, I’ll get like a light-headed – I don’t like that feeling. So, I’ll try to keep it to a minimum and do like two or three puffs at a time. (Case 6, F/29) - “Um, I’ve had – okay experiences– I’m not huge on it because I find that it- it’s very harsh. I know with the, the big rig setup that my ex-boyfriend had, um, you could change the settings to go lower and higher. Um, I find it was just really harsh on my lungs. Um, I felt like I was gonna cough or like – it was, just too much, I guess it’s a different kind of - you know, its vapour instead of a cigarette, but I’m a smoker, so, it was just different for my lungs and I didn’t – I never really got used to it.” (Case 3, F/33) - “I coughed and uh didn’t care for it very much.” (Case 15, F/59) - “Yes, I do get short of breath when I vape.” (Case 15, F/59) |
|  | Negative health effects caused by vaping | Stated negative health effects caused by vaping and/or stated negative effects stopped after stopping vaping | - “So, I, it, it just took some getting used to. Um, I can smoke now, and it’s fine, but like at the beginning I would cough or – um, it would just, um, feel really harsh on my lungs, like too strong.” (Case 3, F/33) - “I found at first, it uh, if I was smoking it too much, I felt nauseous, and I would get headaches. Um, so I slowed down on it. Um, and still if, if I take too many puffs of it, I’ll get like a light-headed – I don’t like that feeling. So, I’ll try to keep it to a minimum and do like two or three puffs at a time.” (Case 6, F/29) - “Cause I heard it was really bad for your, lungs. (Case 2, F/46) - “It suppressed my appetite…” (Case 2, F/46) - “Um, well I started to feel kind of short of breath” (Case 2, F/46) - “I find that it burns my lungs” (Case 29, F/53) - “Yeah, the more you vape, the more it burns.” (Case 29, F/53) - “Hmm, not really, um, just that it’s not as good for you as people think it is.” (Case 2, F/46) - “So there are times where like I notice uh, that like the night before, if I vape like twice as much, or whatever, than I usually do, and I wake up the next morning and it’s like I feel it, it’s like oh shit, I vaped too much last night. (Case 27, F/39) - “I guess? Like that first inhale, when you first wake up and it’s like, and its like [inhales] uhhh – oouuu, like tenderness, almost? Almost like you pulled a muscle? Or something? Maybe not that extreme, but like you overused a muscle but instead it’s your lungs. And there is almost like a hungover feeling as well, like, like uh, like gross taste in your mouth, or like, something, like a grogginess.” (Case 27, F/39) - “Well definitely your lungs right, anything you put into your lungs is not going to be healthy for you, so.” (Case 17, M/41) |
|  | Vaping is addictive | Comments discussing vaping as addictive or potentially addictive, and/or descriptions of the addictive attributes of vaping when explaining their experiences. | - “Well, for-first of all, it was a – a so-social thing and then I started to, to - like it.” (Case 2, F/46) - “Other than that, I feel, it feels pretty good because it helped me quit smoking and I didn’t need the vape all day. And also, I – I – I did not get physically addicted to the vape, and I’m not saying no one, no one else can either, but I don’t know.” (Case 12, F/46) - “So, I had to start to, kinda like, be more mindful of it, and kinda be like okay I’m gonna leave it in another room, I’m gonna like, uh, not – allow myself to not vape in this room. Cause, in my house, for example, um, I would just – they’re’d be like no boundary, I would just sit there and vape. So I had to, at one point, be more mindful of how much I was vaping, um, for some reason once I went from 6 to 3, that – I noticed, it got a lot easier for me, it was almost like that, um, I don’t know if you’ve ever smoked or or not, but when you have like, a nicotine craving, you kinda like “nic” out, like it-it, you get like this anxiety or like this tightness, a lot of people get like, a temper, makes you just kind of snap.” (Case 27, F/39) - “It definitely could be addictive, especially for people who, 1 haven’t smoked anything before and they’re just picking up vaping just because they want to um […] It definitely gets addictive in that scenario, um also for um people who are um cigarette smokers, it would be addictive in the sense that you are already kind of addicted to that in the nicotine so yeah” (Case 14, F/18) |
|  | Vaping can be dangerous | Comments discussing vaping as dangerous due to the substances within vaping liquids or components. | - “Um, actually, one thing is, um, those cartridges, I don’t know what it – I don’t know if uh, there’re different materials like they make out of, but like, the cartridges I buy, are glass, and the ones that I’ve gotten before were actually like a really hard, uh plastic material, and I was using it, and it actually started to bubble. And it almost looked like it was about to burst on me, so – that is kind of iffy of getting one that isn’t glass and it getting high heated, and it bursting on you and maybe blinding you or hurting you somehow.” (Case 26, M/33)) - “So, yeah, some of the vials, they’re glass, right, and then some are plastic and uh, the plastic ones – if you’re using it a lot, uh like I said, a bubble had formed, started like molding and almost like bursting, and yeah, that’s uh, what happened to me." (Case 26, M/33) |
| 11. No perceived impact of vaping on MOUD | Vaping does not impact MOUD | Comments describing neutral/no effect of vaping on MOUD treatment. | - “Uh, no. I, uh – if it does, I haven’t noticed it…” (Case 5, M/25) - “Um, no, never, never did any, never effected it at all, I don’t think.” (Case 12, F/46) - “Uh, never really thought of it, I smoked before I ever started any kind of opiates and I continued smoking through, so…” (Case 13, M/36) - “N-n-no, no, no, no interaction. It’s a totally – apples and oranges. For uh – […] Or fruits and vegetables, no-no-no, no bearing, one doesn’t have any bearing on the other.” (Case 19, M/64) |
| 12. Vaping has some effects on MOUD | Vaping has some effects on MOUD | Comments describing positive effects on MOUD, and indirect benefits of vaping on MOUD treatment success. | - “I find that it makes the methadone seem to last longer.” (Case 29, F/53) - “Right. Like you’ll get the stomach cramps or whatever – The-the vaping will take that away.” (Case 29, F/53) - “If I um, end up missing my drink or end up throwing it up or something, then the metha-or the weed can help subside some of that stuff. Cause I’ll start to feel sick and my legs’ll start to hurt –“ (Case 23, M/28) - "So it doesn’t stop it but it kinda helps and just kinda easing my mind to try not to think about it and” (Case 23, M/28) - “Um I think it’s helped me decrease my methadone.” (Case 32, F/51) - “…a lot of people find the same thing as me that it’s really complimentary. And um, weed fills in the cracks where methadone is not perfect cause no medication can be perfect, right?” (Case 36, N/24) |
| 13. Perception that vaping is for the youth | Vaping is popular among young people | Discussion of vape as something that is most common within a younger age group. | - “Like a little younger, youths, and even uh, students, and I find, uh, their, uh, like a lot of younger people are using it rather than older people, right?” (Case 5, M/25) - “Yeah, like, I can’t name, I can’t name anybody that I know that is underage that vapes, but, I uh, I could imagine, just because of all the different flavours and stuff… that, uh, yeah, I don’t know.” (Case 5, M/25) - “The only thing I’ve heard is that the young kids, the teenagers, they, they get into the, they vape around with no nicotine in it, or something, I don’t know. My kids don’t do that. But, um. So I don’t know, I don’t know if it. That’s it. I don’t know if it’s a negative thing or, if there’s any nicotine in it, I don’t know if does anything for them or not. You know.” (Case 12, F/46) - “Um, but again, I-I don’t even know – cause I know they wanna change the flavours, because they’re like “oh kids are smoking” – like a lot of kids are vaping now. And to that I’d like to say, like, I started smoking in high school, I started smoking cigarettes in high school, so – I mean I don’t know if more kids are vaping than kids started smoking in high school – I think that would be an interesting thing to kind of research.” (Case 27, F/39) - “That kind of high school group. Is doing that. Where, they’re not even smoking cigarettes, they’re just buying these things to smoke ‘em, which – that I can kinda understand, where the government’s coming from, with all the flavours and everything” (Case 28, M/30) |
|  | Vaping flavours are attracting youth | Discussion of how vaping flavour options are attractive to young people, including flavour options without nicotine or cannabis components. | - Yeah, like, I can’t name, I can’t name anybody that I know that is underage that vapes, but, I uh, I could imagine, just because of all the different flavours and stuff… that, uh, yeah, I don’t know (Case 5, M/25) - “Um, I think that kid’s need to be more aware of it, I’ve seen 11-year-olds out there vaping like candy flavours and stuff…” (Case 17, M/41) - “So, it’s – as a quitting tool, it’s amazing. But, I do definitely, that’s why I’m super hyped to help with any research. Cause, like, I wanna give - see what’s going on and learn, especially with like I say, with people who have been picking it up because their friend’s doing it. Or because it tastes good, or you know.” (Case 28, M/30) |
| 14. Vaping to get high | Vaping to get “high” | Comments related to vaping in order to achieve a “high” or feeling of euphoria, typically discussed in the context of cannabis vaping. | - “Well I felt that I could control my high more through vaping.” (Case 34; F/69) - “Um, they just said that I um, tend to like weed, I should give shatter a try. Um, and they said I could use less of it and get more high. So I tried his pen and then I liked it so I went out and grabbed one of mine and I’ve been smoking it ever since.” (Case 23, M/28) - “So, I’ll just vape it, like just to get high. And it will be like a hit here and there.” (Case 7, M/33) |

Participant information is given as (Case No., Gender/Age)

M: Male; F: Female; N: Non-binary; MOUD: Medication for Opioid Use Disorder
