# Supplementary material for: Effects of vaping on physical and mental health in at-risk populations (VAPE): mixed-methods study of motivations for and perspectives on vaping in patients with opioid use disorder
Source: BJPsych Open. 2025 Apr 2;11(3):e75. doi: 10.1192/bjo.2025.6 (PMC12052573; doi:10.1192/bjo.2025.6)
Supplement: D’Elia et al. supplementary material 7 — D’Elia et al. supplementary material [file S2056472425000067sup007.pdf]

Vaping in At-risk Populations: Effects on Mental and Physical Health (VAPE) Study: A Patient Perspective

Study Protocol

Dr. Zainab Samaan, Dr. Lehana Thabane, Dr. Leonora Regenstreif, Dr. Tea Rosic, Dr. Claire de Oliveira, Dr. Parameswaran Nair, Dr. David Marsh, Dr. Laura O'Neill, Nitika Sanger, Alessia D'Elia, Kevin Park

MCMASTER UNIVERSITY

Table of Contents

ADMINISTRATIVE INFORMATION 3

1.0 TITLE 3

2.0 PROTOCOL VERSION 3

3.0 SOURCES OF FUNDING 3

4.0 STUDY TEAM ROLES AND RESPONSIBILITIES 3

TABLE 1 STUDY INVESTIGATORS AND CONTRIBUTORS 3

5.0 INTRODUCTION 4

5.1 BACKGROUND AND RATIONALE 4

6.1 STUDY OBJECTIVES 6

7.0 STUDY METHODS 6

8.0 STUDY SETTING 6

9.1 ELIGIBILITY CRITERIA 6

9.2 RESEARCH PERSONNEL CONDUCTING QUALITATIVE INTERVIEWS 7

10.0 OUTCOMES 7

PRIMARY OUTCOME MEASURES 7

SECONDARY OUTCOME MEASURES 7

11.0 SAMPLE SIZE 7

12.0 RECRUITMENT 7

13.0 BLINDING 7

14.0 DATA COLLECTION METHODS 7

15.0 DATA MANAGEMENT 8

16.0 STATISTICAL ANALYSES 8

17.0 DATA MONITORING 8

18.0 HARMS 8

19.0 AUDITING 9

20.0 RESEARCH ETHICS APPROVAL **9**

21.0 PROTOCOL AMENDMENTS **9**

22.0 CONSENT **9**

23.0 CONFIDENTIALITY **9**

24.0 DECLARATION OF INTERESTS **9**

25.0 ACCESS TO DATA **10**

26.0 DISSEMINATION **10**

27.0 APPENDICES **10**

Study PROTOCOL

ADMINISTRATIVE INFORMATION

1.0 Title
Vaping in At-risk Populations: Effects on Mental and Physical Health (VAPE) Study: A Patient Perspective

2.0 Protocol version
Date and version: Version 1, September 30, 2020

3.0 Sources of funding,
This study is funded by Canadian Institutes for Health Research, HEV-172884 .

4.0 Study Team Roles and Responsibilities
Table 1 below shows the study team members and their specific roles in the study.

Table 1 Study Investigators and Contributors

Individual	Expertise
Scientific team	
Zainab Samaan, MBChB, MRCPsych, PhD	Extensive background in clinical psychiatry and research design; primary investigator for CIHR funded RCT of behavioural intervention for depression, trial methodologist with focus in mental health, clinical professor and collaborator on over 100 publications.
Lehana Thabane, PhD	Internationally renowned epidemiologist and biostatistician with decades of experience in >100 national and international RCTs, leading > 700 peer-reviewed publications published in topic clinical journals that include JAMA, Lancet, BMJ, and Annals of Internal Medicine.
David Marsh, MD	Extensive background in addictions research, chief medical director for Canadian Addiction Treatment Centres and professor of clinical sciences at the Northern Ontario School of Medicine (NOSM).
Parameswaran Nair, MD, PhD, FRCP, FRCPC	Trained and practicing respirologist with extensive research in respiratory health, specifically investigating types of bronchitis and possible novel targeted therapies; Frederick E. Hargreave Teva Innovation Chair in Airway Diseases. Professor of medicine and collaborator on over 100 publications in journals including the NEJM and Lancet.
Leonora Regenstreif, MSc MD, CCFP(AM) FCFP ,MScCH (AMH)	Expertise in primary mental health care, addiction medicine and hospitalized patients with OUD and medical comorbidities related to drug use. Research collaborator in the management of OUD in incarcerated populations.

Tea Rosic, MD, $PhD_{(c)}$	Senior Psychiatry resident and Clinician Investigator Program trainee; PhD student in Health Research Methods with clinical and research experience in substance use disorders.
Laura O'Neill MSW, PhD, RSW, RP	Actively involved in teaching, training and supervising healthcare professionals at McMaster University. BSc (Psychology), a BSW, MSW and a PhD (Social Work). Registered Social Worker with the OCSWSSW, a Registered Psychotherapist with CRPO and a certified CBT therapist with the Canadian Association of Cognitive and Behavioural Therapies.
Nitika Sanger, $PhD_{(c)}$	PhD. Candidate in Medical Science Graduate Program with extensive background in addiction and mental health research.
Alessia D'Elia $PhD_{(c)}$	PhD. Candidate in Neuroscience Graduate Program with extensive background in mental health research.
Kevin Park	Lived experience with vaping behaviour who will contribute to the study design, the development of relevant study questions and support the dissemination of study results.

5.0 INTRODUCTION

5.1 Background and Rationale

Vaping has gained popularity in Canadians, particularly in youth and young adults. Accordingly, many questions have been raised about the short- and long-term health outcomes given the scarcity of current evidence. Regarded by some as an alternative to combustible cigarettes[1], evidence suggests that vaping and e-cigarettes are not as innocuous as this perspective may imply. The use of these products results in the release of harmful toxins that pose important risks to the user, as well as to the recipients of second- and third-hand smoking.[2,3] However, little research has investigated the short- and long-term effects that vaping may have on mental and physical health vulnerabilities. This lack of data is met with an even greater uncertainty of the effects of vaping cannabis products, the investigation of which is made especially important by the recent legalization of cannabis. Research is needed to investigate short and long-term health outcomes of vaping in vulnerable populations, specifically in those receiving out-patient treatment for opioid use disorders (OUD) who commonly use multiple substances[4,5], which may complicate or influence opioid treatment outcomes in these patients.

The number of individuals between the ages of 16 and 19 who report ever having vaped increased from 29.3% to 37.0% between 2017 and 2018, with a corresponding increase in number of days vaped in the same time.[6] While proponents of vaping might suggest increases can be explained by increased uptake of vaping as a substitution for cigarettes in an attempt to quit smoking, evidence indicates that the upward trend in vaping is observed most strongly among those who identify as "never" or "experimental" smokers.[6] As vaping substances such as nicotine or cannabis retains their addictive properties, this behaviour presents a concern for at-risk populations, including those with OUD.

OUD is a serious, chronic, relapsing and remitting characterised, at times, by compulsive use of opioids, and sustained behavioural changes affecting the individual's life and functioning.[7] OUD leads to deleterious consequences to individuals and society, including increased likelihood for infections such as hepatitis and HIV, psychiatric comorbidities, social adverse consequences, and increased mortality.[8–15] Canada is the world's second largest opioid consumer globally[16] and the increased availability of prescribed opioids has contributed to diversion and, in turn, the

current opioid epidemic.[17] Patients with OUD are at greater risk of polysubstance use, as well as physical and mental health co-morbidities.[18,19] Both cigarette smoking[20,21] and cannabis use[22] are highly prevalent in this population and it remains important to examine the impact of vaping on these patients. OUD may also pose a respiratory risk due to the depressive effects of opioids on the respiratory system and additional substance use through insufflation and inhalation, potentially putting these individuals at higher risk of infection and death from the novel COVID-19. Research has suggested that vaping may be associated with respiratory health conditions including chronic obstructive pulmonary disease (COPD) and wheezing.[23,24] The CDC has termed any respiratory condition related to vaping as e-cigarette or vaping product-use associated lung injury (EVALI). EVALI includes any shortness of breath, lung infection, fever, etc. related to vaping. As of January 2020, over 2,700 cases of EVALI have presented in the United States.[25]

Recent research suggests that those who vape may be at increased risk for COVID-19 infection and its more severe consequences. The symptomology associated with COVID-19 includes shortness of breath, fever, and cough, which can lead to pneumonia and acute hypoxemic respiratory failure.[26] There are concerns that smokers, which include those who vape, may develop serious complications if they contract COVID-19.[27] Individuals may also contract COVID-19 through the sharing of vape devices as the coronavirus may remain active on aerosols (and associated surfaces) from several hours to days.[28,29] Additionally, individuals with OUD are also at higher risk of deleterious consequences of COVID-19.[30]

Vaping has also been associated with psychological and mental health concerns. In a study of university students, the use of e-cigarettes (devices that turns liquid containing nicotine into vapor) was found to be associated with multi-drug use, including alcohol and opioids, low self-esteem, symptoms of attention-deficit/hyperactivity disorder, post-traumatic stress disorder, gambling disorder, and anxiety.[31] E-cigarette use was associated with traits of impulsivity[31] and internalizing and externalizing problems.[32] There is likely a bidirectional association between vaping and mental health. Studies showed higher depression and stress scores were predictive of e-cigarette use in college students.[33] These findings highlight the co-occurrence of vaping with mental health challenges and problematic substance use in non-psychiatric populations. Further, these studies highlight the need for investigating vaping behaviour in a high-risk population, and specifically in the context of the opioid epidemic within the current COVID-19 pandemic where vulnerable groups are expected to bear the brunt of the epidemic within the pandemic.

We completed a systematic review of vaping within OUD population and found that some research has suggested that vaping may be associated with adverse outcomes including increased psychiatric distress[34] and physical health symptoms such as coughing and headaches.[35] However, there is no research examining vaping and COVID-19 related symptoms in the OUD population.

Preliminary data from our study investigating pharmacogenetics of OUD in patients receiving medication-assisted treatment (MAT) (POST study, a CIHR funded cohort study, n=2,360, individuals 16 years and older, 56% men) showed 19.2% of individuals with OUD use vaping, with 74% of participants vaping nicotine, 33% vaping cannabis (THC, CBD, Marijuana and shatter), 11% vaping of both cannabis and nicotine, and 5.5% reporting vaping water or “flavour”.[36] The POST Study collected information pertaining to initiation of opioid use, opioid addiction treatment history, cannabis use, illicit drug use, and physical and mental health symptoms, using a set of questions and validated clinical questionnaires. Specific information pertaining to vaping behaviour has also been collected. Participants were asked

about vaping status (binary variable), and the type of substance that they vape (free-text response). Table 1 describes demographic characteristics and vaping behaviours in a subset of the POST study who reported "yes" to vaping (n=453), stratified by sex.

Further data are needed to understand individuals' perceptions and motivations for vaping in the OUD population. Additionally, given the variability of substance use outcomes in those receiving treatment, it is also important to explore is other substance use (i.e., cocaine, opioids, cannabis, amphetamines and benzodiazepine use) also differ in those that vape and have OUD.

6.1 Study Objectives

The primary objective of this qualitative study is to understand the perceptions and motivations for vaping in the OUD population receiving medication assisted treatment. The secondary objective is to collect specific information pertaining to frequency of use, amount, and vaping substance as well as co-substance use information.

7.0 Study Methods

We will conduct qualitative interviews on the phone to obtain information on the perceptions of vaping and vaping behaviour patterns (i.e., reasons for vaping, type of substance, effect of vaping on health) in the OUD population. With the expertise of a qualitative expert, we have designed an unstructured interview guide (see attached interview guide) that have 8 broad, open-ended questions that will allow the interviews flexibility of asking more tailored questions as the participant shares their thoughts and experiences. The qualitative interviews will be audio recorded and transcribed without any identifying information.

In addition to the qualitative interview, we will also administer a Questionnaire of Vaping Craving (QVC), a short validated questionnaire for assessing vaping beliefs and behaviours[37], and collect demographic details. We will also be collecting urine drug screen results for opioids, cocaine, amphetamines, cannabis and benzodiazepines from medical records for a period of 12 months after study enrolment.

Participants will be provided with a $10.00 Tim Hortons gift card for their participation.

8.0 Study Setting

We will recruit 50 participants attending medication assisted treatment clinics through the Canadian Addiction Treatment Centre (CATC).

9.1 Eligibility Criteria

1. People aged 16 years or older
2. Fulfil DSM-5 criteria for OUD
3. Receiving medication assisted treatment for OUD
4. Currently vape
5. Able to provide informed consent

9.2 Research Personnel conducting qualitative interviews

All research assistants involved in conducting the qualitative interviews will be trained by Laura O'Neill.

10.0 Outcomes

Primary Outcome Measures

As the primary aim is to collect information on perceptions of vaping and will be collected through a qualitative interview, there is no structural outcome. This information will be open ended and collected through the interview guide developed.

Secondary Outcome Measures

Our **secondary outcome** is Questionnaire of Vaping Craving (QVC). This will be administered to participants electronically after the qualitative interview. We will also get information relating to co-substance use for a period of 12 months after study enrolment using their medical records at CATC.

11.0 Sample size

Based on the qualitative literature, it has been suggested that one should only conduct qualitative interviews to the point of saturation, that is until no longer obtaining different perspectives. It has been suggested that for observational studies, between 30-50 participants is the recommendation.[38] The sample size for this study is 50 participants.

12.0 Recruitment

We will recruit from Canadian Addiction Treatment Centres. We will provide the centers with a poster announcement to place in the clinic with the study details and contact information. The Director of Operations will be giving us a list of 3 sites that we will recruit from. Once a site has reached saturation, we will be provided with another site. At any given time, the maximum number of sites that will have ongoing recruiting will be 3. We have provided a template for this poster under recruitment material.

13.0 Blinding

This study is qualitative interview therefore is not possible for participants to be blinded.

14.0 Data Collection Methods

A specifically designed case report forms (CRF) will be used to collect the data using electronic research data capture, REDCap. We will be using REDCap to electronically store the surveys containing the outcome instruments. We will be using the phone to conduct the qualitative interviews. The recorded phone interviews will also be stored on REDCap. The recorded interviews will be kept for 10 years and then safely destroyed.

15.0 Data Management

Data will be entered into research Electronic Data Capture (REDCap) (http://project-redcap.org/). Electronic data will be hosted in the local institution server at McMaster Mac or Joes? with passcode protection and electronic security measures in keeping with institutional policy and privacy regulations. Reports will be generated weekly to check data quality and recruitment progress.

16.0 Statistical analyses

Data for this aim will be analyzed using Nvivo 12 qualitative data analysis software (QSR International).[39] The free-text data will be run through a word frequency query to logically arrange the information and determine the most common words. The word count query will help identify initial patterns in the data; there is evidence that this function improves analytic accuracy when compared to manual qualitative word frequency analyses.

In order to avoid decontextualization of the free-text answers, the minimum number of letters permissible in the word frequency query will be set to four. Any word with a frequency weighting of greater than 0.5% will be coded as a node. A node is a collection of references found in the free-text data that corresponds to a particular theme or word. Words with a word frequency percentage above 0.5% that are related to a similar theme will be grouped in the same node. From this output, we will employ matrix coding queries.[41] The output of a matrix coding query is a chart that displays the number of references coded at each node and the corresponding demographic attributes for each participant.

The thematic data will be presented for the overall population, by sex, gender, type of MAT, age, and ethnicity.

Additionally, with the urine drug screen results from the medical records, we will conduct linear and logistic regressions to see if there are any differences between socio-demographic variables and substance use outcomes in this population. We will need to see the distribution of the outcomes to decide on whether a linear or logistic model will be appropriate.

17.0 Data Monitoring

The main concern for this study data is the non-physical risks associated with a loss of privacy or confidentiality. Procedures have been put into place that are designed to keep your information confidential. The consent form and any study identifiers will be stored securely and separately from the collected information. This study does not require data and safety monitoring board due to the reasons described above in a social/behavioral research type of study.

18.0 Harms

This study is a minimal risk non-pharmaceutical study; however, there may be risks involved. The participant may feel upset or distressed when asked about when asked about vaping

behavior. The research assistant will attend to the participant needs and remove them for the study or skip over certain questions if needed.

19.0 Auditing

The study team will virtually meet regularly to discuss the study progress and review the weekly report of study recruitment, data quality and monitoring.

20.0 Research Ethics Approval

Approval from the Hamilton Integrated Research Ethics Board (HIREB) will be sought prior to commencement of study.

21.0 Protocol Amendments

Any changes to the study protocol will be reported to the HIREB prior to implementation. In addition, any protocol deviations/violations will be reported promptly and a note to file will be kept in the study file.

22.0 Consent

Informed consent will be obtained in two ways. The first will be informed verbal consent that will be recorded on the phone in which the research assistant will thoroughly go through the consent form and the study objectives. This verbal consent will be stored separate from the qualitative interview. Once the participant has completed study, they will go to their CATC clinic, sign the written consent form, and pick up their gift card. This 2 stage consent was done to ensure timely interviews are done when participants call the research office then attend the clinic as they would normally do for clinical care for the written consent to be signed and copy is provided to the participant at that time as well as a token of appreciation for their time.

Participants will be informed that they can request additional time to consider participation when they call on the phone, and can be sent a copy of the consent form to review at their leisure before making a decision on enrolling in the study. If they have any questions, the participant can contact the research assistant and principal investigator through the details provided on the consent form.

23.0 Confidentiality

All study personnel will be trained and monitored regularly in the requirement of participants' confidentiality according to hospital and research ethics board regulations and following good clinical practice guidelines. All research related procedures including data collection and storage will be carried out in secure electronic platform of REDCap. No information about any participants will be shared outside the research team without prior consent unless there are concerns regarding participant health and safety.

24.0 Declaration of Interests

The investigators declare no conflict of interests.

25.0 Access to data

Study investigators will have access to the study data.

26.0 Dissemination

We have taken measures to ensure our study is rapidly made widely available. First, we involved respirologist, addiction medicine specialists, the medical director of CATC and a person with lived experience. This team will aid in disseminating through communication, workshops and presentations with target patient populations.

Results and conclusions will be shared with key populations and healthcare personnel through publications in scientific journals and e-conferences, websites, university newsletters, hospital bulletins, grand rounds, educational events and seminars, physical and virtual information tools, and through collaborations with community partners.

27.0 Appendices

1. Qualitative Interview Guide

2. Questionnaire of Vaping Craving (QVC).

3. Informed Consent Form

References

1. Budney, A. J., Sargent, J. D. & Lee, D. C. Vaping cannabis (marijuana): parallel concerns to e-cigs? *Addiction* **110**, 1699–1704 (2015).

2. Cheng, T. Chemical evaluation of electronic cigarettes. *Tob. Control* **23**, ii11–ii17 (2014).

3. Farsalinos, K. E. *et al.* Nicotine levels and presence of selected tobacco-derived toxins in tobacco flavoured electronic cigarette refill liquids. *Int. J. Environ. Res. Public Health* **12**, 3439–3452 (2015).

4. Luo, C. *et al.* Sociodemographic characteristics of patients with children in a methadone maintenance program: A cross-sectional study. *Harm Reduct. J.* (2019). doi:10.1186/s12954-019-0283-9

5. Shams, I. *et al.* The association between health conditions and cannabis use in patients with opioid use disorder receiving methadone maintenance treatment. *BJPsych Open* **5**, 1–8 (2019).

6. Hammond, D. *et al.* Prevalence of vaping and smoking among adolescents in Canada, England, and the United States: Repeat national cross sectional surveys. *The BMJ* (2019). doi:10.1136/bmj.l2219

7. Association, D.-5 A. P. Diagnostic and statistical manual of mental disorders. *Arlingt. Am. Psychiatr. Publ.* (2013).

8. Organization, W. H. *The world health report 2002: reducing risks, promoting healthy life*. (World Health Organization, 2002).

9. Ezzati, M. *et al.* Selected major risk factors and global and regional burden of disease. *Lancet* **360**, 1347–1360 (2002).

10. Zhang, L., Zhang, D., Chen, W., Zou, X. & Ling, L. High prevalence of HIV, HCV and tuberculosis and associated risk behaviours among new entrants of methadone maintenance treatment clinics in Guangdong Province, China. *PLoS One* **8**, e76931 (2013).

11. Wall, R. *et al.* Social costs of untreated opioid dependence. *J. Urban Heal.* **77**, 688–722 (2000).

12. Brooner, R. K., King, V. L., Kidorf, M., Schmidt, C. W. & Bigelow, G. E. Psychiatric and substance use comorbidity among treatment-seeking opioid abusers. *Arch. Gen. Psychiatry* **54**, 71–80 (1997).

13. Seal, K. H. *et al.* Predictors and prevention of nonfatal overdose among street-recruited injection heroin users in the San Francisco Bay Area, 1998–1999. *Am. J. Public Health* **91**, 1842–1846 (2001).

14. Coutinho, R. A. HIV and hepatitis C among injecting drug users: success in preventing HIV has not been mirrored for hepatitis C. (1998).

15. Hall, W., Bell, J. & Carless, J. Crime and drug use among applicants for methadone maintenance. *Drug Alcohol Depend.* **31**, 123–129 (1993).

16. Board, I. N. C. *Report of the International Narcotics Control Board for 2016*. (United Nations Publications, 2016).

17. Dart, R. C. *et al.* Trends in opioid analgesic abuse and mortality in the United States. *N. Engl. J. Med.* **372**, 241–248 (2015).

18. Fischer, B. *et al.* Illicit opioid use in Canada: comparing social, health, and drug use characteristics of untreated users in five cities (OPICAN study). *J. Urban Heal.* **82**, 250–266 (2005).

19. Sanger, N. *et al.* Association Between Socio-Demographic and Health Functioning Variables Among Patients with Opioid Use Disorder Introduced by Prescription: A Prospective Cohort Study. 623–632 (2018).

20. Clemmey, P., Brooner, R., Chutuape, M. A., Kidorf, M. & Stitzer, M. Smoking habits and attitudes in a methadone maintenance treatment population. *Drug Alcohol Depend.* **44**, 123–132 (1997).

21. Richter, K. P., Gibson, C. A., Ahluwalia, J. S. & Schmelzle, K. H. Tobacco use and quit attempts among methadone maintenance clients. *Am. J. Public Health* **91**, 296 (2001).

22. Zielinski, L. *et al.* Association between cannabis use and methadone maintenance treatment outcomes: an investigation into sex differences. *Biol. Sex Differ.* **8**, 8 (2017).

23. Li, D. *et al.* Association of smoking and electronic cigarette use with wheezing and related respiratory symptoms in adults: cross-sectional results from the Population Assessment of Tobacco and Health (PATH) study, wave 2. *Tob. Control* **29**, 140–147 (2020).

24. Xie, Z., Ossip, D. J., Rahman, I. & Li, D. Use of Electronic Cigarettes and Self-Reported Chronic Obstructive Pulmonary Disease Diagnosis in Adults. *Nicotine Tob. Res.* (2019).

25. Christiani, D. C. Vaping-Induced acute lung injury. (2020).

26. Ahn, D.-G. *et al.* Current status of epidemiology, diagnosis, therapeutics, and vaccines for novel coronavirus disease 2019 (COVID-19). *J. Microbiol. Biotechnol.* **30**, 313–324 (2020).

27. Liu, W. *et al.* Analysis of factors associated with disease outcomes in hospitalized patients with 2019 novel coronavirus disease. *Chin. Med. J. (Engl).* (2020).

28. van Doremalen, N. *et al.* Aerosol and surface stability of SARS-CoV-2 as compared with SARS-CoV-1. *N. Engl. J. Med.* **382**, 1564–1567 (2020).

29. Kampf, G., Todt, D., Pfaender, S. & Steinmann, E. Persistence of coronaviruses on inanimate surfaces and its inactivation with biocidal agents. *J. Hosp. Infect.* (2020).

30. Volkow, N. D. Collision of the COVID-19 and addiction epidemics. (2020).

31. Grant, J. E., Lust, K., Fridberg, D. J., King, A. C. & Chamberlain, S. R. E-cigarette use (vaping) is associated with illicit drug use, mental health problems, and impulsivity in university students. *Ann. Clin. Psychiatry* (2019).

32. Conway, K. P. *et al.* Co-occurrence of tobacco product use, substance use, and mental health problems among youth: Findings from wave 1 (2013–2014) of the population assessment of tobacco and health (PATH) study. *Addict. Behav.* (2018). doi:10.1016/j.addbeh.2017.08.009

33. King, J. L., Reboussin, B. A., Spangler, J., Cornacchione Ross, J. & Sutfin, E. L. Tobacco product use and mental health status among young adults. *Addict. Behav.* (2018). doi:10.1016/j.addbeh.2017.09.012

34. Baldassarri, S. R. *et al.* Electronic cigarette and tobacco use in individuals entering methadone or buprenorphine treatment. *Drug Alcohol Depend.* **197**, 37–41 (2019).

35. Stein, M. D. *et al.* An Open Trial of Electronic Cigarettes for Smoking Cessation Among Methadone-Maintained Smokers. *NICOTINE Tob. Res.* **18**, 1157–1162 (2016).

36. Samaan, Z. *et al.* Pharmacogenetics of Opioid Substitution Treatment. (2018).

37. Dowd, AN, *et al*. Development and validation of the questionnaire of vaping craving. *Nicotine Tob Res*. **21**, 63-70 (2019).

38. (Morse, J. M. (1994). Designing funded qualitative research. In Denizin, N. K. & Lincoln, Y. S., *Handbook of qualitative research* (2nd Ed). Thousand Oaks, CA: Sage.).

39. Castleberry A. NVivo 10 [software program]. Version 10. QSR International; 2012. *American journal of pharmaceutical education*. 2014;78(1).
